# Associations of per- and polyfluoroalkyl substances and heavy metals with blood lipid profiles in a representative sample of Korean adolescents

**DOI:** 10.1186/s12940-024-01144-5

**Published:** 2024-11-22

**Authors:** Youlim Kim, Sanghee Shin, Yunsoo Choe, Jaelim Cho, Changsoo Kim, Su Hwan Kim, Kyoung-Nam Kim

**Affiliations:** 1 Department of Preventive Medicine, Yonsei University College of Medicine, 50-1 Yonsei-Ro, Seoul, Seodaemun-Gu 03722 Republic of Korea; 2 Department of Pediatrics, Hanyang University Guri Hospital, Guri, Republic of Korea; 3 Institute of Environmental Research, Yonsei University College of Medicine, Seoul, Republic of Korea; 4 Institute of Human Complexity and Systems Science, Incheon, Republic of Korea; 5 Department of Information Statistics, Gyeongsang National University, Jinju, Republic of Korea

## Abstract

**Background:**

Previous studies on the associations of per- and polyfluoroalkyl substances (PFASs) and heavy metals with lipid profiles among adolescents have been scarce. We sought to investigate the associations of PFASs and heavy metals with blood lipid levels in a representative sample of Korean adolescents.

**Methods:**

Data from the Korean National Environmental Health Survey (2018–2020) were used. Concentrations of PFASs [perfluorooctanoic acid (PFOA), perfluorooctane sulfonic acid (PFOS), perfluorohexane sulfonic acid, perfluorononanoic acid (PFNA), and perfluorodecanoic acid (PFDeA)], lead, and mercury were measured in serum, whole blood, and urine samples, respectively. Linear regression, Bayesian kernel machine regression (BKMR), and k-means clustering analyses were employed to evaluate the associations between pollutants and lipid levels.

**Results:**

In the linear regression analyses, PFOA levels were associated with higher low-density lipoprotein cholesterol (LDL-C) levels; PFOS with higher total cholesterol (TC) levels; PFNA with higher TC, LDL-C, and non-high-density lipoprotein cholesterol (non-HDL-C) levels; PFDeA with higher TC, LDL-C, non-HDL-C, and high-density lipoprotein cholesterol levels; and mercury with higher TC and non-HDL-C levels. The BKMR analysis revealed that the PFAS and heavy metal mixture was associated with higher LDL-C levels (1.8% increase in LDL-C at the 75th percentile of all PFAS and heavy metal concentrations compared to their median values, 95% credible interval: 0.5, 3.1), primarily driven by the effect of PFDeA. Compared to individuals in the low pollutant exposure cluster (geometric mean levels of PFOA, PFOS, PFHxS, PFNA, PFDeA, lead, and mercury were 2.7 μg/L, 6.2 μg/L, 1.6 μg/L, 0.7 μg/L, 0.4 μg/L, 0.8 μg/dL, and 0.3 μg/L, respectively), those in the high pollutant exposure cluster (5.1 μg/L, 10.7 μg/L, 3.7 μg/L, 1.3 μg/L, 0.6 μg/L, 0.9 μg/dL, and 0.4 μg/L, respectively) demonstrated higher TC levels (2.5% increase in TC, 95% confidence interval: 0.1, 5.0) in the k-means clustering analysis.

**Conclusion:**

Due to the limitations of this study, such as its cross-sectional design, these results should be interpreted cautiously and confirmed in future studies before drawing implications for public health strategies aimed at promoting health during adolescence and later in life.

**Supplementary Information:**

The online version contains supplementary material available at 10.1186/s12940-024-01144-5.

## Background

Lipids play a crucial role in maintaining biological structures, such as cell membranes, and supporting the proper function of physiological systems, including hormonal systems [1]. Poor lipid profiles can induce various adverse health outcomes, particularly atherosclerosis and cardiovascular disease (CVD), the leading cause of death globally [2]. Because atherosclerosis, the initial stage of CVD, can begin before adulthood, disruption of lipid metabolism (resulting in poor lipid profiles) during adolescence can increase the risk of CVD in later stages of life [3]. The prevalence of dyslipidemia (abnormal blood lipid levels) was 21.8% among United States adolescents aged 12–19 years [4], 29.1% among Beijing boys and 23.6% among Beijing girls aged 10–18 years [5], and 24.9% among Korean adolescents aged 10–18 years [6].

Per- and polyfluoroalkyl substances (PFASs) are a group of synthetic chemicals characterized by an alkyl chain with one or more fluorinated carbon atoms. Due to their water- and stain-repellent properties and thermal stability, PFASs have been widely used in consumer and industrial products, such as food packaging materials, nonstick cookware, waterproof fabrics, carpeting, firefighting foams, and metal plating [7]. The epidemiological evidence linking PFAS exposure to metabolic outcomes, such as diabetes, overweight, and obesity, remains insufficient [8]. However, the associations between PFAS exposure and blood lipid levels are widely recognized across various populations [9, 10], including children and adolescents [11].

However, a systematic review found inconsistent and controversial results regarding the association between PFAS exposure and blood lipid levels [9]. Additionally, although potential age-related variability was noted [9], studies focusing on pre-adult populations are scarce.

Heavy metals, such as lead and mercury, are legacy pollutants that persist in the environment and have bio-accumulative properties [12]. Humans are exposed to these metals through both anthropogenic sources (e.g., industrial, agricultural, and household activities) and natural sources [10, 11]. A limited number of studies have explored the associations between lead and mercury exposure and lipid levels in the general population, and the results have been inconsistent regarding exposure and outcome parameters [13–18]. In addition, studies investigating these associations in pre-adult populations are even more limited [17, 18].

People are often exposed to multiple pollutants simultaneously, rather than to specific individual pollutants. Sources of co-exposure to PFASs and heavy metals, such as fish and seafood, are estimated to contribute up to 86% of dietary PFAS exposure and are considered an important source of heavy metals, especially mercury [19, 20]. In addition, overlapping biological mechanisms, such as oxidative stress-related hepatotoxicity and perturbation of thyroid and sex hormones, have been suggested for the impacts of PFASs and heavy metals on lipid levels [21–26]. Therefore, synergistic effects and/or concentration addition from co-exposure to PFASs and heavy metals may occur due to these potentially shared toxicological pathways [25, 26]. However, while several studies have investigated the combined effects of PFASs and heavy metals on various outcomes [27–29], to the best of our knowledge, none have examined their combined effects on blood lipid levels, which represents a significant research gap.

Adolescence is considered a vulnerable period for environmental hazards due to immature biological systems, significant physiological and hormonal changes, and rapidly evolving behaviors and the living environments [28, 29]. Therefore, in the present study, we investigated the associations of PFASs and heavy metals with blood lipid levels in a representative sample of Korean adolescents. We hypothesize that PFASs and heavy metals are both individually and collectively associated with unfavorable lipid profiles in adolescents.

## Methods

### Study design and population

This study utilized data from the Korean National Environmental Health Survey (KoNEHS), a nationwide cross-sectional survey conducted by the Korean Ministry of Environment under Article 14 of the Environmental Health Act, 2008. Since 2009, the KoNEHS has been conducted every three years to gather data on environmental pollutant exposure, along with demographic, socioeconomic, and behavioral characteristics in a representative sample of the Korean population. The survey uses a two-stage proportionally stratified sampling design based on region, type of educational institution, age, and gender. It includes biospecimen sampling and analysis, clinical tests, and questionnaires on lifestyle and exposure sources [30, 31]. The survey protocol was reviewed and approved by the Research Ethics Committee of the National Institute of Environmental Research (No. NIER-2018-BR-003–02), and written informed consent was obtained from all participants. This study, using publicly available de-identified data from the KoNEHS, was approved by the Institutional Review Board of Severance Hospital (No. 4–2024-0588).

Since PFASs were first measured in the fourth cycle (2018–2020) of the KoNEHS, and this cycle’s data is the latest publicly available, we utilized it for our study. The adolescent dataset included 828 individuals aged 12–17 years from 67 middle and high schools across the country. After excluding three participants due to the lack of information on blood lipid parameters and seven due to the lack of information on urine mercury levels, the final sample consisted of 818 adolescents. No adolescent reported current use of lipid-lowering medications.

### Biospecimen collection and measurement of PFASs and heavy metals

Biospecimens (blood and urine) were collected at medical institutions near the schools where the participating adolescents were enrolled, without considering fasting status. Samples were transported to biobanks within 24 h while maintaining a temperature of 2–6 ℃. They were then processed and stored in deep freezers at -20 ℃. Aliquots were sent to a certified laboratory for analysis every two weeks.

Detailed descriptions of the analytical methods and quality control procedures for PFASs and heavy metals have been published elsewhere [32–34]. Briefly, 800 μL of acetonitrile was added to a 200 μL of serum sample, followed by centrifugation at 13,000 rpm for 10 min. After 800 μL of supernatant was collected, it was concentrated for 15–20 min using nitrogen gas and reconstituted with 25 μL of 50% methanol. The reconstituted samples were used for quantifying five legacy PFASs [perfluorooctanoic acid (PFOA), perfluorooctane sulfonic acid (PFOS), perfluorohexane sulfonic acid (PFHxS), perfluorononanoic acid (PFNA), and perfluorodecanoic acid (PFDeA)] by high-performance liquid chromatography with tandem mass spectrometry (Q-Sight Triple Quad, PerkinElmer, MA, USA). The limits of detection (LOD) were 0.050 μg/L for PFOA, 0.056 μg/L for PFOS, 0.071 μg/L for PFHxS, 0.019 μg/L for PFNA, and 0.017 μg/L for PFDeA. Two participants had PFHxS levels below the LOD; however, all participants had concentrations above the LOD for the other PFASs.

Blood lead and urine cadmium levels were measured using whole blood and spot urine samples, respectively, by graphite furnace-atomic absorption spectroscopy (AAnalyst 800, PerkinElmer, MA, USA). Urine mercury levels were analyzed using spot urine samples with a gold amalgamation direct mercury analyzer (DMA-80, Milestone, CT, USA). The LOD were 0.17 μg/dL for blood lead, 0.04 μg/L for urine mercury, and 0.04 μg/L for urine cadmium. Although one adolescent had blood lead levels below the LOD and six adolescents had urine mercury levels below the LOD, a substantial proportion of participants (213 adolescents, > 25%) had urine cadmium levels below the LOD. Therefore, we only considered blood lead and urine mercury levels as exposures in further analyses. Urine creatinine levels were measured in spot urine samples used to measure urine mercury levels, employing the Jaffe reaction method (ADVIA 1800 Auto Analyzer, Siemens Healthineers, PA, USA).

Concentrations of PFASs and heavy metals below the LOD were imputed with the LOD value divided by the square root of two [35]. Various quality control measures, including internal quality control programs and participation in the German External Quality Assessment Scheme, were implemented according to the National Institute of Environmental Research guidelines to ensure the precision and accuracy of the analysis.

### Analysis of blood lipid parameters and definition of dyslipidemia

Serum concentrations of total cholesterol (TC), high-density lipoprotein cholesterol (HDL-C), and triglyceride (TG) were measured by the enzymatic method, the elimination/catalase method, and the glycerol phosphate oxidase-Trinder without serum blank method, respectively (ADVIA 1800 Auto Analyzer, Siemens Healthineers, PA, USA). No participant had TC, HDL-C, or TG values lower than the LOD (10.0 mg/dL for TC, 5.0 mg/dL for HDL-C, and 8.0 mg/dL for TG). Serum low-density lipoprotein cholesterol (LDL-C) levels were calculated using the Friedewald equation [36]. We further indirectly estimated non-high-density lipoprotein cholesterol (non-HDL-C) concentrations, a useful screening parameter not affected by fasting status or high TG, by subtracting HDL-C (mg/dL) from TC (mg/dL) [35, 36].

Dyslipidemia was considered as a secondary outcome. According to the National Heart, Lung, and Blood Institute’s Expert Panel Guidelines and the Korean Society of Pediatric Endocrinology’s Clinical Practice Guidelines [35, 37], dyslipidemia was defined as follows: high TC (≥ 200 mg/dL), high LDL-C (≥ 130 mg/dL), high non-HDL-C (≥ 145 mg/dL), low HDL-C (< 40 mg/dL), and high TG (≥ 130 mg/dL).

### Covariates

Based on previous studies [12, 13, 15, 38–42], we constructed a directed acyclic graph to illustrate the potential causal pathway for the associations of PFASs and heavy metals with blood lipid levels (Fig. S1). We identified the following variables as potential confounders or factors influencing lipid levels, without inducing collider stratification bias or mediating the associations: age (years), gender (male or female), body mass index (BMI, kg/m^2^; < 18.5, 18.5–22.9, 23.0–24.9, or ≥ 25.0), paternal educational level (high school or lower, college or university, graduate school, or missing), maternal educational level (high school or lower, college or university, graduate school, or missing), tobacco smoking (never smoker, past smoker, or current smoker), secondhand smoke exposure (no or yes), alcohol consumption (no or yes), regularly engaging in sweat-inducing exercise (no, exercise but not to the point of sweating, or yes), and fish intake (≤ once/month, 2–3 times/month, once/week, or ≥ twice/week). All this information was collected during the survey by trained interviewers using structured questionnaires. Missing values were observed only for paternal (*n* = 16) and maternal educational levels (*n* = 17), for which we used a missing indicator category. To ensure comparability of the results, we adjusted for the same set of variables mentioned above in all analytical models (except for urine creatinine levels, which were additionally included in linear and logistic regression models that considered urine mercury levels as an exposure and in the pollutant mixture analysis models).

### Statistical analysis

To control for the potential impacts of urine dilution on exposure assessment when using urine as a matrix (urine mercury levels), we applied a novel method suggested by O’Brien et al. [43]. The simple standardization method of dividing urine chemical levels by urine creatinine levels may not adequately account for variations in urine creatinine levels due to differences in muscle mass and BMI. To address this, we first predicted urine creatinine levels using linear regression models with age, gender, and BMI as predictors. Thereafter, we divided the measured urine mercury levels by the ratio of measured creatinine levels to predicted creatinine levels. In addition to using the covariate-adjusted standardized exposure measure, we also included urine creatinine levels as a covariate (“covariate-adjusted standardization plus covariate adjustment”) [43].

We applied a natural log transformation to blood lipid parameters (serum TC, LDL-C, non-HDL-C, HDL-C, and TG levels) to approximate normal distributions and reduce the influence of outliers. PFAS (serum PFOA, PFOS, PFHxS, PFNA, and PFDeA levels) and heavy metal concentrations (blood lead and urine mercury levels) were log2-transformed for the same reason and to improve interpretability by estimating results based on the doubling of exposures.

After visually confirming linear exposure-outcome relationships between pollutants and lipid parameters using generalized additive models (the mgcv package in R software) (Fig. S2), we evaluated the associations of individual PFAS and heavy metal exposures with TC, LDL-C, non-HDL-C, HDL-C, and TG levels (continuous variables; primary outcomes) using linear regression models. Appropriate strata, cluster, and weight variables were incorporated into the models to estimate associations representative of Korean adolescent population. We used the SURVEYREG procedure in SAS software for these analyses.

Pearson’s correlation analysis was performed on log-transformed PFASs and heavy metals. Given the observed correlations among pollutants [ρ = 0.08 to 0.85 and all *p*-values < 0.05 except for the correlation between PFOS and lead (ρ = 0.07, *p*-value = 0.06) and between PFHxS and lead (ρ = 0.02, *p*-value = 0.54)] (Table S1) and the potential overlap in the biological mechanisms of their health impacts, we investigated the associations between a chemical mixture consisting of PFASs and heavy metals and blood lipid levels using Bayesian kernel machine regression (BKMR) and k-means clustering methodologies [43, 44].

In the BKMR analysis, which flexibly models non-linear exposure–response relationships and interactions among exposures [44], we evaluated the cumulative overall associations between the chemical mixture and lipid levels by predicting outcomes based on simultaneous increases or decreases in all the considered chemicals from their median values. A Gaussian distribution was applied for modeling blood lipid levels in the BKMR models. We also estimated the posterior inclusion probabilities (PIPs) for each pollutant, which indicate the relative importance of individual exposures in contributing to overall effects. A PIP of > 0.5 suggests that the inclusion of an exposure improves the model fit in more than half of the iterations, making it a meaningful predictor. We then explored the associations between individual chemicals and lipid levels, holding the concentrations of all other chemicals constant at the 25th, 50th, and 75th percentiles, respectively. The BKMR models were adjusted for the same set of covariates and implemented using the Markov Chain Monte Carlo method with 10,000 iterations. A hierarchical variable structure was imposed, grouping all PFASs together and all heavy metals together [27]. The BKMR analyses were conducted using the bkmr package in R software.

To complement the supervised machine learning-based BKMR approach, we also applied the k-means clustering method, a widely used unsupervised dimension reduction technique. In the k-means clustering analysis, we identified actual exposure profiles in this population, clustered adolescents with similar pollutant exposure patterns, and linked these clusters (with distinguishable exposure profiles) to health outcomes. We standardized all PFASs and heavy metals into Z-scores, repeated k-means cluster analyses with increasing numbers of clusters, and determined the optimal number of clusters using the cubic cluster criterion (CCC) and pseudo F statistics. Since the CCC and pseudo F statistics decreased continuously when the number of clusters exceeded two (Table S2), we assigned participants into two clusters [45]. We then constructed similar linear regression models, considering the complex survey design and adjusting for the same covariates, while using cluster membership instead of individual chemical levels as exposures. The cluster with the lower chemical exposure profile served as the reference. These analyses were performed using the STANDARD and FASTCLUS procedures in SAS software.

We also conducted analyses for secondary binary outcomes (high TC, high LDL-C, high non-HDL-C, low HDL-C, and high TG) using logistic regression models (the SURVEYLOGISTIC procedure in SAS software) and BKMR models with a probit link function (the bkmr package in R software). For the logistic regression models, we used individual pollutants, as well as the cluster memberships identified from k-means clustering, as exposures. The covariates and model specifications were consistent with those used for continuous lipid level analyses.

We performed gender-stratified analyses and evaluated the interactions between pollutants and gender by testing the product terms of pollutant levels and gender. These analyses were conducted because previous studies have reported gender-specific differences in the health effects of PFAS and heavy metal exposure [39, 40, 45]. Additionally, disruption of sex hormone homeostasis, such as estrogen imbalance, may be one of the underlying mechanisms for the associations of PFASs and heavy metals with lipid levels [40, 45].

We performed several sensitivity analyses to confirm the robustness of the results. First, we repeated the analyses without adjusting for BMI, considering the potential mediating role of BMI. Second, we adjusted for the consumption of big fish and tuna (≤ once/month, 1–3 times/month, or ≥ once/week) instead of total fish intake. Third, we included frozen meal intake (almost none, 1–3 times/month, once/week, or ≥ twice/week) as an additional covariate to account for the overall diet quality, as a comprehensive diet quality index was not available in the KoNEHS dataset. Fourth, we controlled for urine dilution effects using the conventional standardization method (dividing urine chemical levels by urine creatinine levels) instead of the method suggested by O’Brien et al. [46]. This allowed for direct comparison with previous studies that utilized the conventional standardization method. Fifth, we assessed mercury exposure using blood mercury levels, a commonly used biomarker primarily reflecting methylmercury, instead of urine mercury levels. Blood mercury levels were measured using whole blood samples with a gold amalgamation direct mercury analyzer (DMA-80, Milestone, CT, USA), with a LOD of 0.01 μg/L. One participant had a value below the LOD, which was imputed as the LOD divided by the square root of two. Sixth, we created a categorized variable for the covariate-adjusted standardized urine cadmium levels (below the LOD; greater than or equal to the LOD but below the median; and greater than or equal to the median) and evaluated the associations of urine cadmium levels (categorized variable) with blood lipid levels and dyslipidemia using the same linear and logistic regression models, with the ‘below the LOD’ category as the reference.

We presented the results from linear regressions and BKMR analyses with continuous outcomes as percent (%) changes per doubling of concentrations of PFASs and heavy metals, calculated using the formula: $100\times ({e}^{\upbeta }-1)$, where β represents a regression coefficient. For logistic regressions, results were presented as odds ratios (ORs). Since only probit regressions were available for binary outcomes in the BKMR analysis, we presented regression coefficients from probit regressions (β_probit_) for the dyslipidemia outcomes. To increase interpretability, we also translated β_probit_ into the more familiar association estimator of OR using the formula: $OR\approx e^{1.6\times\beta\text{probit}}$ [47, 48]. All analyses were performed using SAS (version 9.4, SAS Institute Inc.) and R (version 4.3.2, R Development Core Team) software.

## Results

The mean (± standard deviation) age of the study participants was 14.6 (± 1.7) years, with 53.6% being female. A total of 52.3% of participants had a BMI of 18.5–22.9 kg/m^2^, while 20.3% had a BMI of ≥ 25.0 kg/m^2^. The majority of participants had fathers (51.1%) and mothers (50.1%) with a college or university education, were never smokers (94.0%), did not experience secondhand smoke exposure (78.0%), and did not drink alcohol (65.4%). The geometric mean (± geometric standard deviation) concentrations were 3.6 (± 1.6) μg/L for PFOA, 8.0 (± 1.7) μg/L for PFOS, 2.4 (± 2.3) μg/L for PFHxS, 0.9 (± 1.5) μg/L for PFNA, 0.5 (± 1.4) μg/L for PFDeA, 0.8 (± 1.5) μg/dL for lead, and 0.3 (± 2.0) μg/L for mercury (Table 1).

**Table 1:** Characteristics of the study participants by dyslipidemia status

Variables	Total (*n* = 818)	No dyslipidemia (*n* = 554)	High TC (*n* = 36)	High LDL-C (*n* = 12)	High non-HDL-C (*n* = 37)	Low HDL-C (*n* = 85)	High TG (*n* = 209)
Age (year)	14.6 ± 1.7	14.7 ± 1.7	15.2 ± 1.8	15.5 ± 2.0	14.8 ± 1.8	14.6 ± 1.7	14.5 ± 1.7
Gender							
Male	380 (46.5)	239 (43.1)	6 (16.7)	4 (33.3)	12 (32.4)	57 (67.1)	112 (53.6)
Female	438 (53.6)	315 (56.9)	30 (83.3)	8 (66.7)	25 (67.6)	28 (32.9)	97 (46.4)
Body mass index (kg/m^2^)							
< 18.5	107 (13.1)	86 (15.5)	3 (8.3)	0 (0)	1 (2.7)	3 (3.5)	17 (8.1)
18.5–22.9	428 (52.3)	322 (58.1)	15 (41.7)	7 (58.3)	11 (29.7)	30 (35.3)	83 (39.7)
23.0–24.9	117 (14.3)	70 (12.6)	6 (16.7)	3 (25.0)	8 (21.6)	10 (11.8)	36 (17.2)
≥ 25.0	166 (20.3)	76 (13.7)	12 (33.3)	2 (16.7)	17 (46.0)	42 (49.4)	73 (34.9)
Paternal educational level							
≤ High school	308 (37.7)	204 (36.8)	14 (38.9)	5 (41.7)	16 (43.2)	34 (40.0)	75 (35.9)
College or university	418 (51.1)	283 (51.1)	18 (50.0)	6 (50.0)	18 (48.7)	47 (55.3)	113 (54.1)
Graduate school	76 (9.3)	54 (9.8)	4 (11.1)	1 (8.3)	3 (8.1)	4 (4.7)	18 (8.6)
Missing	16 (2.0)	13 (2.4)	0 (0)	0 (0)	0 (0)	0 (0)	3 (1.4)
Maternal educational level							
≤ High school	333 (40.7)	223 (40.3)	16 (44.4)	7 (58.3)	18 (48.7)	35 (41.2)	83 (39.7)
College or university	410 (50.1)	279 (50.4)	15 (41.7)	4 (33.3)	15 (40.5)	41 (48.2)	108 (51.7)
Graduate school	58 (7.1)	42 (7.6)	4 (11.1)	1 (8.3)	3 (8.1)	6 (7.1)	13 (6.2)
Missing	17 (2.1)	10 (1.8)	1 (2.8)	0 (0)	1 (2.7)	3 (3.5)	5 (2.4)
Tobacco smoking							
Never smoker	769 (94.0)	525 (94.8)	36 (100.0)	12 (100.0)	37 (100.0)	77 (90.6)	195 (93.3)
Past smoker	24 (2.9)	13 (2.4)	0 (0)	0 (0)	0 (0)	5 (5.9)	8 (3.8)
Current smoker	25 (3.1)	16 (2.9)	0 (0)	0 (0)	0 (0)	3 (3.5)	6 (2.9)
Secondhand smoke exposure							
No	638 (78.0)	440 (79.4)	21 (58.3)	8 (66.7)	25 (67.6)	62 (72.9)	166 (79.4)
Yes	180 (22.0)	114 (20.6)	15 (41.7)	4 (33.3)	12 (32.4)	23 (27.1)	43 (20.6)
Alcohol consumption							
No	535 (65.4)	358 (64.6)	24 (66.7)	7 (58.3)	26 (70.3)	57 (67.1)	142 (67.9)
Yes	283 (34.6)	196 (35.4)	12 (33.3)	5 (41.7)	11 (29.7)	28 (32.9)	67 (32.1)
Regular exercise							
No	271 (33.1)	190 (34.3)	21 (58.3)	9 (75.0)	19 (51.4)	21 (24.7)	63 (30.1)
Exercise but not to the point of sweating	211 (25.8)	144 (26.0)	6 (16.7)	0 (0)	9 (24.3)	25 (29.4)	56 (26.8)
Sweat-inducing exercise	336 (41.1)	220 (39.7)	9 (25.0)	3 (25.0)	9 (24.3)	39 (45.9)	90 (43.1)
Fish intake							
≤ Once/month	306 (37.4)	202 (36.5)	18 (50.0)	7 (58.3)	17 (46.0)	38 (44.7)	76 (36.4)
2–3 times/month	260 (31.8)	178 (32.1)	9 (25.0)	2 (16.7)	9 (24.3)	23 (27.1)	66 (31.6)
Once/week	142 (17.4)	101 (18.2)	4 (11.1)	1 (8.3)	7 (18.9)	13 (15.3)	37 (17.7)
≥ Twice/week	110 (13.5)	73 (13.2)	5 (13.9)	2 (16.7)	4 (10.8)	11 (12.9)	30 (14.4)
Serum PFOA (μg/L)	3.6 ± 1.6	3.6 ± 1.6	3.3 ± 1.5	4.1 ± 1.5	3.6 ± 1.6	3.7 ± 1.6	3.6 ± 1.5
Serum PFOS (μg/L)	8.0 ± 1.7	7.9 ± 1.7	7.9 ± 1.7	8.7 ± 1.7	8.0 ± 1.7	7.7 ± 1.9	8.1 ± 1.7
Serum PFHxS (μg/L)	2.4 ± 2.3	2.4 ± 2.3	1.8 ± 1.7	2.1 ± 1.7	2.1 ± 1.8	2.6 ± 2.3	2.4 ± 2.2
Serum PFNA (μg/L)	0.9 ± 1.5	0.9 ± 1.5	1.0 ± 1.4	1.1 ± 1.3	1.0 ± 1.6	0.9 ± 1.6	0.9 ± 1.6
Serum PFDeA (μg/L)	0.5 ± 1.4	0.5 ± 1.4	0.4 ± 1.4	0.5 ± 1.3	0.4 ± 1.5	0.4 ± 1.4	0.4 ± 1.5
Blood lead (μg/dL)	0.8 ± 1.5	0.8 ± 1.5	0.7 ± 1.5	0.7 ± 1.4	0.7 ± 1.4	0.8 ± 1.5	0.8 ± 1.5
Urine mercury (μg/L)	0.3 ± 2.0	0.3 ± 2.0	0.3 ± 1.9	0.4 ± 1.6	0.3 ± 1.9	0.3 ± 1.7	0.3 ± 1.9

High TC is defined as TC ≥ 200 mg/dL, high LDL-C as LDL-C ≥ 130 mg/dL, high non-HDL-C as non-HDL-C ≥ 145 mg/dL, low HDL-C as HDL-C < 40 mg/dL, and high TG as TG ≥ 130 mg/dL

Categorical variables are expressed as *n* (%), while continuous variables are represented as the mean ± standard deviation for age, and geometric mean ± geometric standard deviation for per- and polyfluoroalkyl substances and heavy metals. Urine mercury concentrations (μg/L) that are not adjusted for urine creatinine levels are presented

*TC* Total cholesterol, *LDL-C* Low-density lipoprotein cholesterol, *non-HDL-C* Non-high-density lipoprotein cholesterol, *HDL-C* High-density lipoprotein cholesterol, *TG* Triglyceride, *PFOA* Perfluorooctanoic acid, *PFOS* Perfluorooctane sulfonic acid, *PFHxS* Perfluorohexane sulfonic acid, *PFNA* Perfluorononanoic acid, *PFDeA* Perfluorodecanoic acid

Compared to adolescents without any of these conditions (*n* = 554), those with high TC (*n* = 36), high LDL-C (*n* = 12), and high non-HDL-C (*n* = 37) were more likely to be female, have a BMI of ≥ 25.0 kg/m^2^, be exposed to secondhand smoke, and were less likely to exercise regularly. Compared to adolescents without any of these conditions, those with low HDL-C (*n* = 85) and high TG (*n* = 209) were more likely to be male and have a BMI of ≥ 25.0 kg/m^2^ (Table 1).

In the linear regression analyses evaluating one-to-one associations between each pollutant and outcome, a doubling of PFOA levels was associated with a 3.3% [95% confidence interval (CI): 0.2, 6.4] increase in LDL-C levels, and a doubling of PFOS levels was associated with a 2.0% (0.5, 3.4) increase in TC levels. PFNA and PFDeA levels were associated with higher TC [2.9% (0.7, 5.2) for PFNA; 3.9% (1.6, 6.2) for PFDeA], LDL-C [5.3% (1.8, 9.0) for PFNA; 7.6% (3.7, 11.6) for PFDeA], and non-HDL-C levels [3.4% (0.2, 6.7) for PFNA; 4.6% (1.2, 8.1) for PFDeA]. PFDeA levels were also associated with higher HDL-C levels [2.8% (0.2, 5.5)]. A doubling of mercury levels was associated with a 1.6% (0.2, 3.1) increase in TC levels and a 2.5% (0.5, 4.6) increase in non-HDL-C levels (Table 2). In the logistic regression models for dyslipidemia, PFNA and PFDeA levels were associated with higher odds of high LDL-C (OR = 2.6, 95% CI: 1.3, 5.3 for PFNA; OR = 3.1, 95% CI: 1.1, 8.8 for PFDeA) and lower odds of high TG (OR = 0.7, 95% CI: 0.5, 1.0 for PFNA; OR = 0.6, 95% CI: 0.4, 0.9 for PFDeA). Additionally, lead levels were associated with lower odds of high TG (OR = 0.7, 95% CI: 0.5, 0.9) (Table S3).

**Table 2:** Percent changes in blood lipid levels per doubling of concentrations of per- and polyfluoroalkyl substances and heavy metals, estimated from linear regression models

	TC		LDL-C		Non-HDL-C		HDL-C		TG	
	Percent (%) change	95% CI	Percent (%) change	95% CI	Percent (%) change	95% CI	Percent (%) change	95% CI	Percent (%) change	95% CI
PFOA	1.8	-0.3, 3.9	3.3	0.2, 6.4	2.2	-0.8, 5.2	0.8	-1.4, 3.1	-1.8	-7.3, 4.0
PFOS	2.0	0.5, 3.4	1.1	-1.7, 4.0	1.9	-0.1, 3.9	2.0	-0.02, 4.0	3.2	-0.7, 7.2
PFHxS	0.02	-1.0, 1.0	-0.1	-1.9, 1.8	-0.03	-1.6, 1.6	0.02	-1.0, 1.1	0.1	-3.6, 3.9
PFNA	2.9	0.7, 5.2	5.3	1.8, 9.0	3.4	0.2, 6.7	1.9	-0.6, 4.5	-2.8	-8.4, 3.1
PFDeA	3.9	1.6, 6.2	7.6	3.7, 11.6	4.6	1.2, 8.1	2.8	0.2, 5.5	-4.7	-11.3, 2.3
Lead	-0.2	-1.8, 1.5	-1.0	-3.5, 1.7	-1.5	-3.6, 0.7	2.6	-0.2, 5.5	-4.3	-10.5, 2.4
Mercury	1.6	0.2, 3.1	2.4	-0.1, 5.0	2.5	0.5, 4.6	-0.4	-1.8, 1.0	2.0	-2.3, 6.5

The results were estimated from linear regression models with appropriate strata, cluster, and weight variables, adjusted for age, gender, body mass index, paternal educational level, maternal educational level, tobacco smoking, secondhand smoke exposure, alcohol consumption, regular physical exercise, and fish intake. The models that considered urine mercury levels as an exposure were additionally adjusted for urine creatinine levels

*TC* Total cholesterol, *LDL-C* Low-density lipoprotein cholesterol, *non-HDL-C* non-high-density lipoprotein cholesterol, *HDL-C* High-density lipoprotein cholesterol, *TG* Triglyceride, *CI* Confidence interval, *PFOA* Perfluorooctanoic acid, *PFOS* Perfluorooctane sulfonic acid, *PFHxS* Perfluorohexane sulfonic acid, *PFNA* Perfluorononanoic acid, *PFDeA* perfluorodecanoic acid

**Fig. 1:**
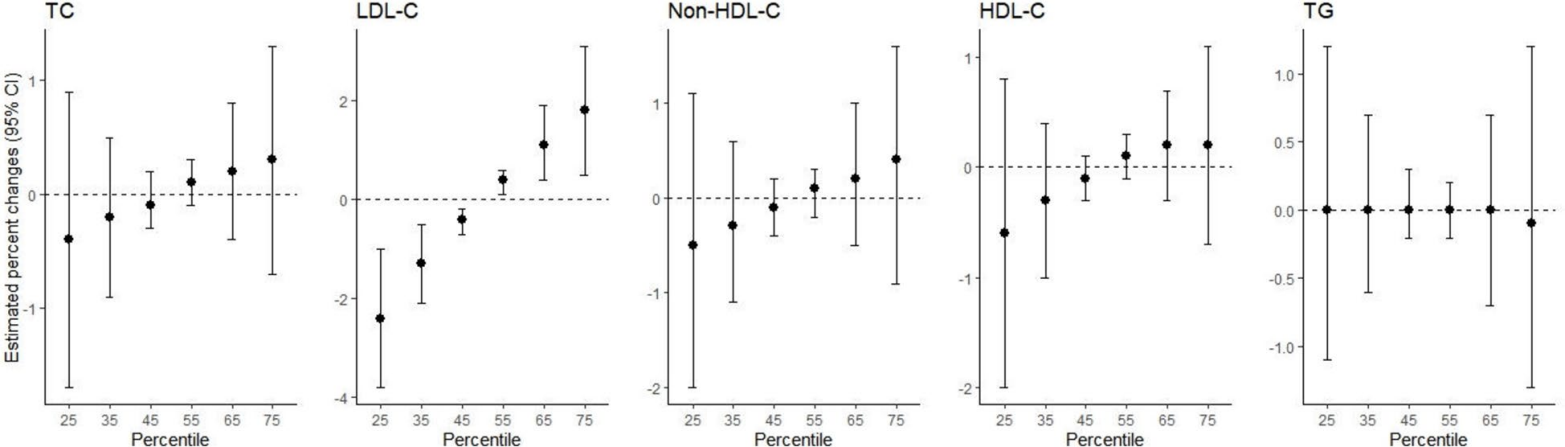
Overall associations between co-exposures to per- and polyfluoroalkyl substances and heavy metals and blood lipid levels, estimated using Bayesian kernel machine regression analyses. Abbreviations: TC, total cholesterol; LDL-C, low-density lipoprotein cholesterol; non-HDL-C, non-high-density lipoprotein cholesterol; HDL-C, high-density lipoprotein cholesterol; TG, triglyceride; CI, confidence interval. Circles represent point estimates of the associations, and error bars indicate 95% confidence intervals. The models were adjusted for age, gender, body mass index, paternal educational level, maternal educational level, tobacco smoking, secondhand smoke exposure, alcohol consumption, regular physical exercise, fish intake, and urine creatinine levels

Co-exposure to PFASs and heavy metals was associated with higher LDL-C levels, but not with other lipid levels or dyslipidemia parameters, in the BKMR analysis (Fig. 1). LDL-C levels were estimated to increase by 1.8% [95% credible interval (CrI): 0.5, 3.1] when concentrations of all PFASs and heavy metals were at the 75th percentile compared to their median values, and to decrease by 2.4% (95% CrI: -3.8, -1.0) at the 25th percentile (Table S4). PFDeA was the only pollutant which had a PIP value of > 0.5 for predicting LDL-C levels (group PIP: 0.97; conditional PIP: 0.98) (Table S5). A doubling of PFDeA levels was associated with a 4.3% increase in LDL-C levels (95% CrI: 1.4, 7.3) after holding the levels of all other pollutants at the 25th, 50th, or 75th percentiles. No other associations were found between pairs of pollutants (PFASs and heavy metals) and outcomes (lipid levels and dyslipidemia) when controlling for all other pollutants (Table S6).

We identified two contrasting clusters from the k-means clustering analysis: one cluster with higher levels of PFASs and heavy metals (*n* = 376) and another with lower levels of PFASs and heavy metals (*n* = 442). The geometric mean (± geometric standard deviation) levels of PFOA, PFOS, PFHxS, PFNA, PFDeA, lead, and mercury were 5.1 (± 1.4) μg/L, 10.7 (± 1.6) μg/L, 3.7 (± 2.3) μg/L, 1.3 (± 1.4) μg/L, 0.6 (± 1.3) μg/L, 0.9 (± 1.4) μg/dL, and 0.4 (± 2.0) μg/L, respectively, in the high pollutant exposure cluster, and 2.7 (± 1.3) μg/L, 6.2 (± 1.5) μg/L, 1.6 (± 1.9) μg/L, 0.7 (± 1.4) μg/L, 0.4 (± 1.3) μg/L, 0.8 (± 1.5) μg/dL, and 0.3 (± 1.9) μg/L, respectively, in the low pollutant exposure cluster (Fig. S3). Compared to adolescents in the low pollutant exposure cluster, those in the high pollutant exposure cluster had 2.5% (95% CI: 0.1, 5.0) higher TC levels. However, no precise associations were found for other lipid levels and dyslipidemia outcomes, as the wider CIs included one (Table 3).

**Table 3:** Associations of a pollutant mixture (high vs. low exposure cluster) with blood lipid levels and dyslipidemia, with clusters identified from k-means clustering analyses

Blood lipid parameter	Lipid levels		Dyslipidemia	
	Percent (%) change	95% CI	OR	95% CI
TC	2.5	0.1, 5.0	0.6	0.3, 1.3
LDL-C	3.6	-0.3, 7.6	1.8	0.6, 5.0
Non-HDL-C	1.9	-1.9, 5.8	0.6	0.2, 1.7
HDL-C	3.6	-0.05, 7.4	0.9	0.5, 1.9
TG	-2.7	-9.9, 5.1	0.7	0.5, 1.2

Dyslipidemia is defined as follows: high TC as TC ≥ 200 mg/dL; high LDL-C as LDL-C ≥ 130 mg/dL; high non-HDL-C as non-HDL-C ≥ 145 mg/dL; low HDL-C as HDL-C < 40 mg/dL; and high TG as TG ≥ 130 mg/dL

Linear and logistic regression models, with appropriate strata, cluster, and weight variables, were used to evaluate the associations of a pollutant mixture (high vs. low exposure cluster) with lipid levels and dyslipidemia, respectively. High (*n* = 376) and low (*n* = 442) exposure clusters were identified from k-means clustering analyses. Linear and logistic models were adjusted for age, gender, body mass index, paternal educational level, maternal educational level, tobacco smoking, secondhand smoke exposure, alcohol consumption, regular physical exercise, and fish intake

*CI* Confidence interval, *OR* Odds ratio, *TC* Total cholesterol, *LDL-C* Low-density lipoprotein cholesterol, *non-HDL-C* non-high-density lipoprotein cholesterol, *HDL-C* High-density lipoprotein cholesterol, *TG* Triglyceride

**Fig. 2:**
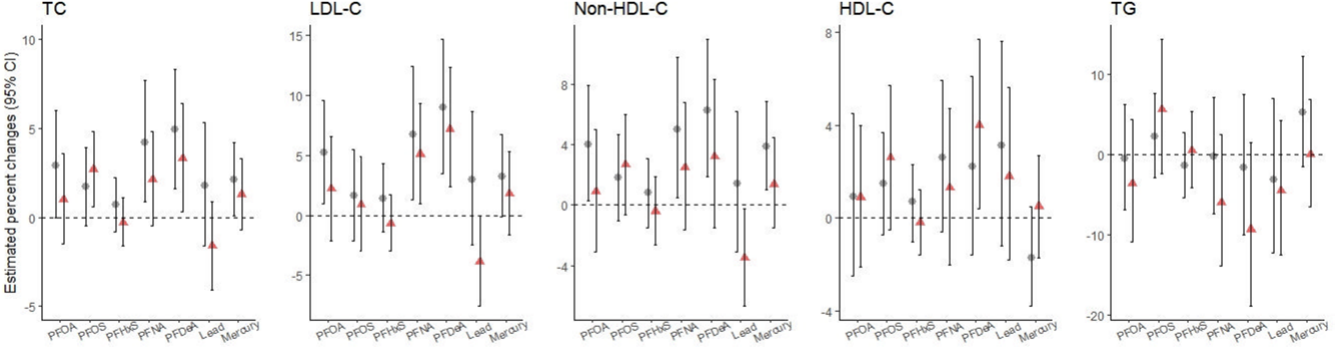
Associations of per- and polyfluoroalkyl substances and heavy metals with blood lipid levels, stratified by gender. Abbreviations: TC, total cholesterol; LDL-C, low-density lipoprotein cholesterol; non-HDL-C, non-high-density lipoprotein cholesterol; HDL-C, high-density lipoprotein cholesterol; TG, triglyceride; CI, confidence interval; PFOA, perfluorooctanoic acid; PFOS, perfluorooctane sulfonic acid; PFHxS, perfluorohexane sulfonic acid; PFNA, perfluorononanoic acid; PFDeA, perfluorodecanoic acid. Gray circles and red triangles represent estimated percent changes in blood lipid levels per doubling of pollutant concentrations among boys and girls, respectively. Error bars indicate 95% confidence intervals. The results were estimated from linear regression models with appropriate strata, cluster, and weight variables, adjusted for age, body mass index, paternal educational level, maternal educational level, tobacco smoking, secondhand smoke exposure, alcohol consumption, regular physical exercise, and fish intake. The models that considered urine mercury levels as an exposure were additionally adjusted for urine creatinine levels

The point estimates of the associations between pollutants and lipid levels were generally greater in boys than in girls, although statistically significant interactions between pollutants and gender were not found (*p*-value for interaction ≥ 0.05), except for the interaction between lead and gender regarding LDL-C levels (*p*-value for interaction = 0.04). The point estimates of the associations between lead levels and LDL-C levels were positive in boys [3.0% (-2.5, 8.7)] and negative in girls [-3.9% (-7.8, -0.004)] (Fig. 2; Table S7).

The following sensitivity analyses were conducted: The results were robust with minimal change in the analysis not adjusted for BMI (Table S8), the analysis adjusted for the intake of big fish and tuna instead of total fish intake (Tables S9), and the analysis additionally adjusted for frozen meal intake (Table S10). The results were also consistent in the analysis that controlled for urine dilution effects using the conventional standardization method (Table S11), as well as in the analysis that used blood mercury levels as exposures (Table S11). We did not find any associations between urine cadmium levels and blood lipid levels or dyslipidemia (Table S11).

## Discussion

This study was conducted with a representative sample of Korean adolescents, whose PFAS and heavy metal levels were comparable to or slightly higher than those found in adolescents from the United States and European countries [29, 49–52]. In the linear regression analyses evaluating one-to-one associations, PFOA and PFOS levels were associated with higher LDL-C and TC levels, respectively. PFNA and PFDeA levels were associated with higher levels of TC, LDL-C, and non-HDL-C, with PFDeA also linked to higher HDL-C levels. Mercury was associated with higher TC and non-HDL-C levels. BKMR analyses indicated that co-exposure to PFASs and heavy metals was associated with higher LDL-C levels, primarily due to the independent effect of PFDeA. K-means clustering analyses revealed that adolescents in the high pollutant exposure cluster had higher TC levels compared to those in the low pollutant exposure cluster. The point estimates of the associations between pollutants and lipid levels were generally greater in boys than in girls, although a statistically significant interaction was found only between lead and gender regarding LDL-C levels.

A systematic review of seven cross-sectional studies and five cohort studies conducted among children and adolescents found that PFASs, particularly PFOS, were associated with higher TC and LDL-C levels, but not with HDL-C or TG levels [11]. While we identified some associations not supported by previous studies (e.g., the associations of PFDeA with higher TC, LDL-C, non-HDL-C, and HDL-C levels) [3, 4], the results of the previous studies and our study suggest that PFAS exposure may lead to higher TC and LDL-C levels, which can increase the risk of CVD [2]. Meanwhile, studies on the associations between heavy metal exposure and lipid profiles are relatively scarce, especially among adolescents, although the findings of existing studies generally align with those of the present study [53–55]. For example, in a cross-sectional study conducted among United States adolescents aged 12–19 years, total blood mercury and methyl mercury levels were reportedly associated with higher TC levels, but not with LDL-C, HDL-C, or TG levels [53]. Further discussions are presented in the Supplementary Material.

Although PFASs and heavy metals share common exposure sources (such as dietary factors, including fish) [19, 20] and potentially overlapping biological mechanisms [25, 26], to our knowledge, the present study is the first to explore the overall associations between co-exposure to PFASs and heavy metals and lipid levels. In the present study, the BKMR analysis revealed that the observed association between the PFAS and heavy metal mixture and LDL-C levels was primarily driven by PFASs, particularly PFDeA, rather than by heavy metals. Additionally, the k-means clustering analysis showed that adolescents in the cluster with higher levels of PFASs and heavy metals had higher TC levels compared to those in the cluster with lower levels of these substances. This may be explained by the strong discrimination of PFOS levels across high and low pollutant exposure clusters (Fig. S3), considering the association between PFOS and TC identified in the single-pollutant linear regression models (Table 2). While BKMR analysis estimates overall effects by predicting outcomes based on hypothetical simultaneous increases in all considered exposures [49], k-means clustering analysis identifies existing exposure profiles in the data and explores the health effects associated with these profiles [56]. Therefore, these two approaches capture slightly different aspects of mixture effects and can be used complementarily. Furthermore, LDL-C constitutes a major portion of TC, and the direction of the associations with TC and LDL-C was found to be the same in both BKMR and k-means clustering analyses, despite differences in the precision of the estimators (Tables S4 and 3).

Among the PFASs and heavy metals examined in this study, PFDeA was found to be associated with LDL-C levels, even when controlling for other pollutants in the BKMR analysis. Owing to its resistance to water, stains, and heat, PFDeA has been widely used for decades in products such as furniture, carpets, outdoor textiles, cosmetics, and paper food containers [57]. In a study conducted in the Boston area of the United States, plasma PFDeA concentrations, but not those of PFOA, PFOS, PFHxS, and PFNA, were associated with higher TC and LDL-C levels in mid-childhood (median age of 7.7 years) [58]. In another study exploring the associations between 12 PFASs and cardiometabolic markers among Arizona firefighters aged 49–54 years, only serum PFDeA levels were associated with blood lipid levels, specifically lower TC levels [59]. A study performing untargeted metabolomic profiling using blood samples (573 lipid and amino acid metabolites) reported that PFDeA levels were associated with lipid metabolites among children aged 6 years, after correction for multiple comparisons [60]. The results of the present study and previous studies suggest that PFDeA may be a key contributor to the observed association between PFAS exposure and lipid levels. However, further studies are warranted to confirm these findings and inform policy implications, given the high correlations among substances (e.g., PFASs), which complicate the disentangling of individual pollutant impacts and contribute to the instability of the results.

In this study, the point estimates of the associations between PFAS and heavy metal levels and lipid levels were generally greater in boys than in girls. We postulate that this result may be explained by the protective effects of estrogen on lipid metabolism [61], which could reduce the deleterious effects of pollutants on lipid profiles in girls. However, although the impacts of endogenous estrogen on lipid metabolism and the related differences in dyslipidemia and CVD risk by gender are well established in epidemiological and mechanistic literature [61], previous studies have reported heterogeneous results regarding gender differences in the associations between PFAS and heavy metal exposures and lipid levels. Some studies found more pronounced associations between PFAS and heavy metal exposures and lipid levels in boys [11, 12], while other studies reported stronger associations in girls [4, 8]. These variations could be due to different patterns of confounding structures, selection bias, and random error. Because investigating the heterogeneity of the association by gender can shed light on the underlying mechanisms and this issue has not been fully explored, further research is needed to confirm the results while addressing concerns of residual confounding, selection bias, and random error.

Several possible biological mechanisms can be proposed for the findings of this study. Both PFAS and heavy metal exposures can induce oxidative stress-related hepatotoxicity, which may disrupt hepatic lipid metabolism and increase the risk of poor lipid profiles [13, 62]. In addition, both PFASs and heavy metals reportedly can affect the endocrine system and disrupt the homeostasis of thyroid and sex hormones, which in turn can alter lipid levels [23–26]. The potential overlapping mechanisms of the impacts of PFAS and heavy metal exposures on lipid levels raise the possibility of synergistic effects and/or concentration addition for toxicity [18, 19], underscoring the need for mixture analysis to accurately estimate the overall effects of co-exposure to these pollutants. Furthermore, PFASs may influence the activation of peroxisome proliferator-activated receptor alpha, a nuclear receptor involved in regulating lipid metabolism in the liver [63], leading to changes in lipid levels [64].

Following limitations need to be considered in the present study. First, due to the cross-sectional study design and the inherent concern of temporal ambiguity between exposures and outcomes, the results of this study cannot be interpreted to establish causal relationships between the considered pollutants and lipid profiles. Second, the analyses were performed only on adolescents aged 12–17 years, because PFAS and lead levels were not measured in children under 12 years in the fourth cycle of the KoNEHS. However, a previous systematic review suggested that the associations between PFAS exposure and lipid levels (TC and LDL-C) may be more prominent among adolescents than among younger children, possibly due to relatively higher ingestion and bioaccumulation of PFAS in the adolescent group [11]. Third, the participants of the present study, who were between 12 and 17 years old, may have been at different pubertal stages, which could influence lipid levels [65]. However, we could not account for these stages, as this information was not provided in the KoNEHS data. Fourth, although legacy PFASs have been gradually replaced by alternative (short-chain fluorinated or non-fluorinated) PFASs and concerns about these alternatives are growing, we considered only five legacy PFASs (PFOA, PFOS, PFHxS, PFNA, and PFDeA) due to data availability in the KoNEHS. Additionally, previous studies have reported associations between various metals, including essential metals (e.g., copper, manganese, selenium, and zinc), and lipid levels [12, 13]. However, we evaluated the health effects of only lead and mercury due to data limitations. Furthermore, although fish is a common exposure source for various pollutants, including dioxins and polychlorinated biphenyls [66], we could not evaluate the effects of these other pollutants on lipid levels due to the lack of information in the KoNEHS data. Future studies should consider a broader range of PFASs, heavy metals, and other pollutants to more accurately reflect real-world exposure patterns, which are continually changing. Fifth, dietary factors can be an important source for PFASs and heavy metals and can affect lipid levels as well, potentially acting as confounders. However, comprehensive information on dietary factors was not available, and a concern about residual confounding by dietary factors remains (e.g., total energy intake, overall diet quality, and omega-3 polyunsaturated fatty acids), although we adjusted the analyses for fish intake, consumption of big fish and tuna, and frozen meal intake [48]. Sixth, given the numerous analyses conducted in this study, some observed associations may be false positives resulting from inflated alpha error caused by multiple testing. Consequently, these results should be interpreted with caution, taking this limitation into account.

However, the present study also has strengths to be acknowledged. First, to our knowledge, this is the first study to explore the overall associations between co-exposure to PFASs and heavy metals and lipid levels. In addition, although adolescents are considered a vulnerable population to environmental pollutants [28, 29], research on the health effects of environmental pollutants, including PFASs and heavy metals, conducted among adolescents is relatively limited compared to that conducted among other older (e.g., adults aged ≥ 65 years) and younger (e.g., infants, preschool-age children, and school-age children) vulnerable populations. Second, we leveraged high-quality nationwide data representative of Korean adolescents, collected under strict survey protocols and quality control procedures [59, 60]. The use of the KoNEHS data allowed us to increase the validity, reliability, and generalizability of the results by minimizing the possibility of exposure and outcome misclassification, confounding bias, and selection bias. Third, we employed two mixture analysis methodologies that can capture different aspects of mixture effects: the BKMR analysis, which is a supervised machine-learning method, and the k-means clustering analysis, which is an unsupervised dimension reduction method. These two distinct methodologies can act complementarily, offering further insights into the impacts of pollutants of interest on lipid profiles in realistic scenarios.

## Conclusions

PFASs and heavy metals were both individually and collectively associated with unfavorable lipid profiles in a representative sample of Korean adolescents. Specifically, in the linear regression analyses, PFOA levels were associated with higher LDL-C levels; PFOS with higher TC levels; PFNA with higher TC, LDL-C, and non-HDL-C levels; PFDeA with higher TC, LDL-C, non-HDL-C, and HDL-C levels; and mercury with higher TC and non-HDL-C levels. The PFAS and heavy metal mixture was associated with higher LDL-C levels, primarily driven by the effect of PFDeA, in the BKMR analysis. Adolescents in the high pollutant exposure cluster showed higher TC levels compared to those in the low pollutant exposure cluster in the k-means clustering analysis. Poor lipid profiles during adolescence, a common health problem that is increasing in many parts of the world, can elevate the risk of various diseases, including CVD, in adulthood. Due to the limitations of this study, such as its cross-sectional design, the results should be interpreted cautiously and confirmed by future studies before drawing implications for public health strategies aimed at reducing the burden of various diseases, including CVD, and promoting health during adolescence and later in life.

## Supplementary Information

Supplementary Material 1.

## Data Availability

Data are available from the authors upon reasonable request.
