# Supplementary material for: Associations of per- and polyfluoroalkyl substances and heavy metals with blood lipid profiles in a representative sample of Korean adolescents
Source: Environ Health. 2024 Nov 22;23:104. doi: 10.1186/s12940-024-01144-5 (PMC11583531; doi:10.1186/s12940-024-01144-5)
Supplement: Supplementary file 1 — Supplementary Material 1. [file 12940_2024_1144_MOESM1_ESM.docx]

*Supplementary Material*

**Associations of per- and polyfluoroalkyl substances and heavy metals with blood lipid profiles in a representative sample of Korean adolescents**

**Table of Contents**

[**Discussion** 1](#_Toc182330434)

[**Table S1.** Correlations among concentrations of per- and polyfluoroalkyl substances and heavy metals in adolescents from the Korea National Environmental Health Survey 3](#_Toc182330435)

[**Table S2.** The cubic cluster criterion and pseudo F statistics according to the number of clusters in the k-means clustering analysis 4](#_Toc182330436)

[**Table S3.** Odds ratios for dyslipidemia per doubling of concentrations of per- and polyfluoroalkyl substances and heavy metals, estimated from logistic regression models 5](#_Toc182330437)

[**Table S4.** Percent changes in blood lipid levels due to co-exposures to per- and polyfluoroalkyl substances and heavy metals, estimated using Bayesian kernel machine regression analyses 7](#_Toc182330438)

[**Table S5.** Group and conditional posterior inclusion probabilities for per- and polyfluoroalkyl substances and heavy metals in the Bayesian kernel machine regression analysis 9](#_Toc182330439)

[**Table S6.** Associations of concentrations of individual per- and polyfluoroalkyl substances and heavy metals with blood lipid levels while holding the concentrations of all other chemicals constant at the 25th, 50th, and 75th percentiles in the Bayesian kernel machine regression analyses 11](#_Toc182330440)

[**Table S7.** Percent changes in blood lipid levels per doubling of concentrations of per- and polyfluoroalkyl substances and heavy metals, estimated from linear regression models stratified by gender 14](#_Toc182330441)

[**Table S8.** Percent changes in blood lipid levels per doubling of concentrations of per- and polyfluoroalkyl substances and heavy metals, estimated from linear regression models not adjusted for body mass index 17](#_Toc182330442)

[**Table S9.** Percent changes in blood lipid levels per doubling of concentrations of per- and polyfluoroalkyl substances and heavy metals, estimated from linear regression models adjusted for the intake of big fish and tuna instead of total fish intake 19](#_Toc182330443)

[**Table S10.** Percent changes in blood lipid levels per doubling of concentrations of per- and polyfluoroalkyl substances and heavy metals, estimated from linear regression models additionally adjusted for frozen meal intake 21](#_Toc182330444)

[**Table S11.** Associations of urine mercury, blood mercury, and urine cadmium levels with blood lipid levels 23](#_Toc182330445)

[**Fig. S1.** Assumed causal pathway for the impacts of per- and polyfluoroalkyl substance and heavy metal exposures on blood lipid levels. Abbreviation: PFASs, per- and polyfluoroalkyl substances. 25](#_Toc182330446)

[**Fig. S2.** Exposure-outcome relationships between per- and polyfluoroalkyl substances and heavy metals and blood lipid levels, estimated using generalized additive models. 26](#_Toc182330447)

[**Fig. S3.** Distributions of concentrations of per- and polyfluoroalkyl substances and heavy metals in high and low pollutant exposure clusters, as identified from the k-means clustering analysis. 28](#_Toc182330448)

[**References** 29](#_Toc182330449)

# **Discussion**

*The associations between individual per- and polyfluoroalkyl substances (PFASs), heavy metals, and lipid profiles*

A recent systematic review of epidemiological studies conducted among general populations provided evidence of associations between perfluorooctanoic acid (PFOA), perfluorooctane sulfonic acid (PFOS), and perfluorononanoic acid (PFNA) levels and higher total cholesterol (TC) levels, as well as associations between PFOS and perfluoroundecanoic acid levels and higher low-density lipoprotein cholesterol (LDL-C) levels [1]. This review also suggested that these associations may be modified by age and highlighted the lack of adjustment for confounders, such as tobacco smoking and dietary factors, in many studies [1]. The present study, conducted among an adolescent population and adjusted for tobacco smoking, secondhand smoke exposure, and dietary factors such as fish intake, consumption of big fish and tuna, and frozen meal intake, addressed the issues raised by the systematic review. Another systematic review of 7 cross-sectional studies and 5 cohort studies conducted among children and adolescents found that PFASs, particularly PFOS, were associated with higher TC and LDL-C levels, but not with high-density lipoprotein cholesterol (HDL-C) or triglyceride (TG) levels [2]. While we identified additional associations not supported by the two systematic reviews [e.g., the associations of perfluorodecanoic acid (PFDeA) with higher TC, LDL-C, non-high-density lipoprotein cholesterol (non-HDL-C), and HDL-C levels] [3, 4], collectively, the results of the two reviews and our study suggest that PFAS exposure may lead to higher TC and LDL-C levels, which can increase the risk of cardiovascular disease (CVD) [3]. These findings reinforce global initiatives to regulate PFAS, starting with PFOS and PFOA and expanding to other PFASs [4].

Previous studies on the associations between lead and mercury exposure and lipid profiles are relatively scarce, especially among adolescents [5–7]. The findings of most previous studies align with the results of the present study. For example, in a cross-sectional study conducted among United States adolescents aged 12–19 years, total blood mercury and methyl mercury levels were reportedly associated with higher TC levels, but not with LDL-C, HDL-C, or TG levels [5]. In another cross-sectional study conducted in the United States among adolescents aged 13–19 years, blood mercury levels were associated with higher TC levels but not with LDL-C or TG levels, while blood lead levels were not associated with any of the lipid profile parameters considered (TC, LDL-C, and TG) [7]. However, a cross-sectional study conducted among Iranian adolescents (mean age of 15 years) reported that blood mercury levels were associated with higher TC and TG levels but not with LDL-C or HDL-C levels in girls, whereas in boys, mercury levels were associated only with higher TG levels [6]. The discrepancies in the results may stem from differences in population characteristics, analytical strategies, and statistical methods, selection and confounding biases, and random error. Further research among pre-adult populations, including adolescents, is needed to draw conclusions about the causal effects of heavy metals on lipid levels.

# **Table S1.** Correlations among concentrations of per- and polyfluoroalkyl substances and heavy metals in adolescents from the Korea National Environmental Health Survey

|  | PFOA | PFOS | PFHxS | PFNA | PFDeA | Lead | Mercury |
| --- | --- | --- | --- | --- | --- | --- | --- |
| PFOA |  | 0.37  (< 0.0001) | 0.54  (< 0.0001) | 0.81  (< 0.0001) | 0.71  (< 0.0001) | 0.16  (< 0.0001) | 0.15  (< 0.0001) |
| PFOS |  |  | 0.28  (< 0.0001) | 0.46  (< 0.0001) | 0.43  (< 0.0001) | 0.07  (0.06) | 0.13  (0.0003) |
| PFHxS |  |  |  | 0.38  (< 0.0001) | 0.33  (< 0.0001) | 0.02  (0.54) | 0.08  (0.02) |
| PFNA |  |  |  |  | 0.85  (< 0.0001) | 0.24  (< 0.0001) | 0.25  (< 0.0001) |
| PFDeA |  |  |  |  |  | 0.20  (< 0.0001) | 0.31  (< 0.0001) |
| Lead |  |  |  |  |  |  | 0.10  (0.0095) |

Abbreviations: PFOA, perfluorooctanoic acid; PFOS, perfluorooctane sulfonic acid; PFHxS, perfluorohexane sulfonic acid; PFNA, perfluorononanoic acid; PFDeA, perfluorodecanoic acid.

The values are presented as ρ (*p*-value).

# **Table S2.** The cubic cluster criterion and pseudo F statistics according to the number of clusters in the k-means clustering analysis

| The number of clusters | Cubic cluster criterion | Pseudo F statistics |
| --- | --- | --- |
| 2 | 66.7 | 342.0 |
| 3 | 48.5 | 234.5 |
| 4 | 45.7 | 208.8 |
| 5 | 37.7 | 177.9 |
| 6 | 31.4 | 158.0 |
| 7 | 27.4 | 146.5 |
| 8 | 25.0 | 140.2 |
| 9 | 23.6 | 130.4 |

# **Table S3.** Odds ratios for dyslipidemia per doubling of concentrations of per- and polyfluoroalkyl substances and heavy metals, estimated from logistic regression models

|  | High TC | | High LDL-C | | High non-HDL-C | | Low HDL-C | | High TG | |
| --- | --- | --- | --- | --- | --- | --- | --- | --- | --- | --- |
|  | OR | 95% CI | OR | 95% CI | OR | 95% CI | OR | 95% CI | OR | 95% CI |
| PFOA | 0.8 | 0.5, 1.5 | 2.0 | 0.9, 4.7 | 1.1 | 0.5, 2.2 | 0.8 | 0.5, 1.3 | 0.8 | 0.7, 1.1 |
| PFOS | 1.4 | 0.8, 2.3 | 1.7 | 0.9, 3.2 | 1.3 | 0.9, 1.8 | 1.0 | 0.7, 1.5 | 1.1 | 0.9, 1.3 |
| PFHxS | 0.9 | 0.7, 1.2 | 1.2 | 0.8, 2.0 | 1.0 | 0.7, 1.4 | 1.1 | 0.9, 1.4 | 1.0 | 0.8, 1.1 |
| PFNA | 1.2 | 0.8, 2.0 | 2.6 | 1.3, 5.3 | 1.1 | 0.5, 2.4 | 0.7 | 0.5, 1.1 | 0.7 | 0.5, 1.0 |
| PFDeA | 1.1 | 0.5, 2.4 | 3.1 | 1.1, 8.8 | 1.0 | 0.4, 2.4 | 0.8 | 0.5, 1.3 | 0.6 | 0.4, 0.9 |
| Lead | 0.7 | 0.4, 1.1 | 0.6 | 0.3, 1.1 | 0.6 | 0.4, 1.0 | 0.7 | 0.4, 1.2 | 0.7 | 0.5, 0.9 |
| Mercury | 1.1 | 0.7, 1.7 | 1.7 | 0.7, 3.8 | 1.1 | 0.7, 1.7 | 1.0 | 0.7, 1.4 | 1.1 | 0.8, 1.4 |

Abbreviations: TC, total cholesterol; LDL-C, low-density lipoprotein cholesterol; non-HDL-C, non-high-density lipoprotein cholesterol; HDL-C, high-density lipoprotein cholesterol; TG, triglyceride; OR, odds ratio; CI, confidence interval; PFOA, perfluorooctanoic acid; PFOS, perfluorooctane sulfonic acid; PFHxS, perfluorohexane sulfonic acid; PFNA, perfluorononanoic acid; PFDeA, perfluorodecanoic acid.

High TC is defined as TC ≥ 200 mg/dL, high LDL-C as LDL-C ≥ 130 mg/dL, high non-HDL-C as non-HDL-C ≥ 145 mg/dL, low HDL-C as HDL-C < 40 mg/dL, and high TG as TG ≥ 130 mg/dL.

The results were estimated from logistic regression models with appropriate strata, cluster, and weight variables, adjusted for age, gender, body mass index, paternal educational level, maternal educational level, tobacco smoking, secondhand smoke exposure, alcohol consumption, regular physical exercise, and fish intake. The models that considered urine mercury levels as an exposure were additionally adjusted for urine creatinine levels.

# **Table S4.** Percent changes in blood lipid levels due to co-exposures to per- and polyfluoroalkyl substances and heavy metals, estimated using Bayesian kernel machine regression analyses

|  | Percent (%) change | 95% credible interval |
| --- | --- | --- |
| TC |  |  |
| 25th percentile of exposure | -0.4 | -1.7, 0.9 |
| 75th percentile of exposure | 0.3 | -0.7, 1.3 |
| LDL-C |  |  |
| 25th percentile of exposure | -2.4 | -3.8, -1.0 |
| 75th percentile of exposure | 1.8 | 0.5, 3.1 |
| Non-HDL-C |  |  |
| 25th percentile of exposure | -0.5 | -2.0, 1.1 |
| 75th percentile of exposure | 0.4 | -0.9, 1.6 |
| HDL-C |  |  |
| 25th percentile of exposure | -0.6 | -2.0, 0.8 |
| 75th percentile of exposure | 0.2 | -0.7, 1.1 |
| TG |  |  |
| 25th percentile of exposure | 0.03 | -1.1, 1.2 |
| 75th percentile of exposure | -0.1 | -1.3, 1.2 |

Abbreviations: TC, total cholesterol; LDL-C, low-density lipoprotein cholesterol; non-HDL-C, non-high-density lipoprotein cholesterol; HDL-C, high-density lipoprotein cholesterol; TG, triglyceride.

The results were estimated when the concentrations of all pollutants (per- and polyfluoroalkyl substances and heavy metals) were at the 25th and 75th percentiles, compared to their median values. The models were adjusted for age, gender, body mass index, paternal educational level, maternal educational level, tobacco smoking, secondhand smoke exposure, alcohol consumption, regular physical exercise, fish intake, and urine creatinine levels.

# **Table S5.** Group and conditional posterior inclusion probabilities for per- and polyfluoroalkyl substances and heavy metals in the Bayesian kernel machine regression analysis

| Pollutants | TC | | LDL-C | | Non-HDL-C | | HDL-C | | TG | |
| --- | --- | --- | --- | --- | --- | --- | --- | --- | --- | --- |
|  | PIP1 | PIP2 | PIP1 | PIP2 | PIP1 | PIP2 | PIP1 | PIP2 | PIP1 | PIP2 |
| PFOA | 0.31 | 0.03 | 0.97 | 0 | 0.30 | 0.04 | 0.50 | 0.52 | 0.06 | 0.21 |
| PFOS | 0.31 | 0.10 | 0.97 | 0 | 0.30 | 0.01 | 0.50 | 0.06 | 0.06 | 0.29 |
| PFHxS | 0.31 | 0 | 0.97 | 0 | 0.30 | 0 | 0.50 | 0 | 0.06 | 0.01 |
| PFNA | 0.31 | 0.12 | 0.97 | 0.02 | 0.30 | 0.10 | 0.50 | 0.40 | 0.06 | 0.17 |
| PFDeA | 0.31 | 0.75 | 0.97 | 0.98 | 0.30 | 0.85 | 0.50 | 0.03 | 0.06 | 0.32 |
| Lead | 0 | 0.40 | 0 | 1 | 0.01 | 0.53 | 0.01 | 0.90 | 0.06 | 0.89 |
| Mercury | 0 | 0.60 | 0 | 0 | 0.01 | 0.47 | 0.01 | 0.10 | 0.06 | 0.11 |

Abbreviations: TC, total cholesterol; LDL-C, low-density lipoprotein cholesterol; non-HDL-C, non-high-density lipoprotein cholesterol; HDL-C, high-density lipoprotein cholesterol; TG, triglyceride; PIP, posterior inclusion probability; PFOA, perfluorooctanoic acid; PFOS, perfluorooctane sulfonic acid; PFHxS, perfluorohexane sulfonic acid; PFNA, perfluorononanoic acid; PFDeA, perfluorodecanoic acid.

PIP1 and PIP2 represent group and conditional PIP, respectively.

# **Table S6.** Associations of concentrations of individual per- and polyfluoroalkyl substances and heavy metals with blood lipid levels while holding the concentrations of all other chemicals constant at the 25th, 50th, and 75th percentiles in the Bayesian kernel machine regression analyses

|  | All other pollutants at 25th percentile | | All other pollutants at 50th percentile | | All other pollutants at 75th percentile | |
| --- | --- | --- | --- | --- | --- | --- |
|  | Percent (%) change | 95% CrI | Percent (%) change | 95% CrI | Percent (%) change | 95% CrI |
| TC | | | | | | |
| PFOA | 0.0 | -0.3, 0.3 | 0.0 | -0.3, 0.3 | 0.0 | -0.3, 0.3 |
| PFOS | 0.1 | -0.8, 1 | 0.1 | -0.8, 1 | 0.1 | -0.8, 1 |
| PFHxS | – | – | – | – | – | – |
| PFNA | 0.1 | -0.6, 0.8 | 0.1 | -0.6, 0.8 | 0.1 | -0.6, 0.8 |
| PFDeA | 0.6 | -1.5, 2.8 | 0.6 | -1.5, 2.8 | 0.6 | -1.5, 2.8 |
| Lead | – | – | – | – | – | – |
| Mercury | 0.0 | -0.2, 0.2 | 0.0 | -0.2, 0.2 | 0.0 | -0.2, 0.2 |
| LDL-C | | | | | | |
| PFOA | – | – | – | – | – | – |
| PFOS | – | – | – | – | – | – |
| PFHxS | – | – | – | – | – | – |
| PFNA | 0.1 | -0.9, 1 | 0.1 | -0.9, 1 | 0.1 | -0.9, 1 |
| PFDeA | 4.3 | 1.4, 7.3 | 4.3 | 1.4, 7.3 | 4.3 | 1.4, 7.3 |
| Lead | 0.0 | -0.2, 0.2 | 0.0 | -0.1, 0.1 | 0.0 | -0.1, 0.1 |
| Mercury | – | – | – | – | – | – |
| Non-HDL-C | | | | | | |
| PFOA | 0.0 | -0.5, 0.5 | 0.0 | -0.5, 0.5 | 0.0 | -0.5, 0.5 |
| PFOS | 0.0 | -0.2, 0.3 | 0.0 | -0.2, 0.3 | 0.0 | -0.2, 0.3 |
| PFHxS | – | – | – | – | – | – |
| PFNA | 0.1 | -0.8, 0.9 | 0.1 | -0.8, 0.9 | 0.1 | -0.8, 0.9 |
| PFDeA | 0.8 | -1.9, 3.5 | 0.8 | -1.9, 3.5 | 0.8 | -1.9, 3.5 |
| Lead | 0.0 | -0.2, 0.2 | 0 | -0.2, 0.2 | 0.0 | -0.2, 0.2 |
| Mercury | – | – | – | – | – | – |
| HDL-C | | | | | | |
| PFOA | 0.4 | -1.2, 1.9 | 0.4 | -1.2, 1.9 | 0.4 | -1.2, 1.9 |
| PFOS | 0.1 | -0.9, 1.1 | 0.1 | -0.9, 1.1 | 0.1 | -0.9, 1.1 |
| PFHxS | – | – | – | – | – | – |
| PFNA | 0.3 | -1.2, 1.8 | 0.3 | -1.2, 1.8 | 0.3 | -1.2, 1.8 |
| PFDeA | 0.0 | -0.4, 0.4 | 0.0 | -0.4, 0.4 | 0.0 | -0.4, 0.4 |
| Lead | 0.0 | -0.3, 0.4 | 0.0 | -0.3, 0.4 | 0.0 | -0.3, 0.4 |
| Mercury | 0.0 | -0.1, 0.1 | 0.0 | -0.1, 0.1 | 0.0 | -0.1, 0.1 |
| TG | | | | | | |
| PFOA | 0.0 | -0.5, 0.5 | 0.0 | -0.5, 0.5 | 0.0 | -0.5, 0.5 |
| PFOS | 0.1 | -1.1, 1.2 | 0.1 | -1.1, 1.2 | 0.1 | -1.1, 1.2 |
| PFHxS | 0.0 | -0.2, 0.2 | 0.0 | -0.2, 0.2 | 0.0 | -0.2, 0.2 |
| PFNA | 0.0 | -0.5, 0.5 | 0.0 | -0.5, 0.5 | 0.0 | -0.5, 0.5 |
| PFDeA | 0.0 | -0.8, 0.8 | 0.0 | -0.8, 0.8 | 0.0 | -0.8, 0.8 |
| Lead | -0.1 | -1.7, 1.4 | -0.1 | -1.7, 1.4 | -0.1 | -1.7, 1.4 |
| Mercury | 0.0 | -0.5, 0.5 | 0.0 | -0.5, 0.5 | 0.0 | -0.5, 0.5 |

Abbreviations: TC, total cholesterol; LDL-C, low-density lipoprotein cholesterol; non-HDL-C, non-high-density lipoprotein cholesterol; HDL-C, high-density lipoprotein cholesterol; TG, triglyceride; CrI, credible interval; PFOA, perfluorooctanoic acid; PFOS, perfluorooctane sulfonic acid; PFHxS, perfluorohexane sulfonic acid; PFNA, perfluorononanoic acid; PFDeA, perfluorodecanoic acid.

The results were presented as percent (%) changes per doubling of chemical concentrations. The models were adjusted for age, gender, body mass index, paternal educational level, maternal educational level, tobacco smoking, secondhand smoke exposure, alcohol consumption, regular physical exercise, fish intake, and urine creatinine levels.

# **Table S7.** Percent changes in blood lipid levels per doubling of concentrations of per- and polyfluoroalkyl substances and heavy metals, estimated from linear regression models stratified by gender

| Pollutant | Gender | TC | | LDL-C | | Non-HDL-C | | HDL-C | | TG | |
| --- | --- | --- | --- | --- | --- | --- | --- | --- | --- | --- | --- |
|  |  | Percent change (95% CI) | *p*-int | Percent change (95% CI) | *p*-int | Percent change (95% CI) | *p*-int | Percent change (95% CI) | *p*-int | Percent change (95% CI) | *p*-int |
| PFOA | Boy | 2.9  (-0.02, 6.0) | 0.42 | 5.2  (1.0, 9.6) | 0.40 | 4.0  (0.3, 7.9) | 0.32 | 0.9  (-2.5, 4.5) | 0.93 | -0.5  (-6.8, 6.2) | 0.58 |
|  | Girl | 1.0  (-1.5, 3.6) |  | 1.6  (-2.1, 5.5) |  | 0.9  (-3.1, 5.0) |  | 0.9  (-2.1, 4.0) |  | -3.6  (-10.9, 4.4) |  |
| PFOS | Boy | 1.7  (-0.5, 3.9) | 0.75 | 1.6  (-2.1, 5.5) | 0.80 | 1.8  (-1.0, 4.7) | 0.92 | 1.5  (-0.7, 3.7) | 0.47 | 2.2  (-2.8, 7.6) | 0.99 |
|  | Girl | 2.7  (0.6, 4.8) |  | 0.9  (-3.0, 4.9) |  | 2.7  (-0.6, 6.0) |  | 2.6  (-0.5, 5.7) |  | 5.6  (-2.4, 14.4) |  |
| PFHxS | Boy | 0.7  (-0.8, 2.2) | 0.36 | 1.4  (-1.4, 4.3) | 0.32 | 0.8  (-1.5, 3.1) | 0.56 | 0.7  (-1.0, 2.3) | 0.25 | -1.4  (-5.3, 2.7) | 0.50 |
|  | Girl | -0.3  (-1.6, 1.1) |  | -0.7  (-3.0, 1.7) |  | -0.4  (-2.6, 1.9) |  | -0.2  (-1.6, 1.2) |  | 0.5  (-4.1, 5.4) |  |
| PFNA | Boy | 4.2  (0.9, 7.7) | 0.41 | 6.7  (1.3, 12.4) | 0.68 | 5.0  (0.5, 9.8) | 0.39 | 2.6  (-0.6, 5.9) | 0.96 | -0.3  (-7.3, 7.1) | 0.25 |
|  | Girl | 2.1  (-0.5, 4.8) |  | 5.1  (1.0, 9.3) |  | 2.5  (-1.6, 6.8) |  | 1.3  (-2.0, 4.7) |  | -6.0  (-13.8, 2.5) |  |
| PFDeA | Boy | 4.9  (1.6, 8.3) | 0.62 | 9.0  (3.5, 14.7) | 0.72 | 6.3  (1.9, 11.0) | 0.35 | 2.2  (-1.6, 6.1) | 0.17 | -1.6  (-10.0, 7.5) | 0.23 |
|  | Girl | 3.3  (0.3, 6.4) |  | 7.2  (2.4, 12.3) |  | 3.2  (-1.5, 8.3) |  | 4.0  (0.4, 7.7) |  | -9.3  (-18.9, 1.5) |  |
| Lead | Boy | 1.8  (-1.6, 5.3) | 0.11 | 3.0  (-2.5, 8.7) | 0.04 | 1.4  (-3.1, 6.2) | 0.09 | 3.1  (-1.2, 7.6) | 0.71 | -3.1  (-12.2, 7.0) | 0.84 |
|  | Girl | -1.6  (-4.1, 0.9) |  | -3.9  (-7.8, -0.004) |  | -3.5  (-6.7, -0.2) |  | 1.8  (-1.8, 5.6) |  | -4.5  (-12.5, 4.3) |  |
| Mercury | Boy | 2.1  (0.1, 4.2) | 0.33 | 3.2  (-0.1, 6.7) | 0.42 | 3.9  (1.0, 6.9) | 0.21 | -1.7  (-3.8, 0.5) | 0.56 | 5.2  (-1.5, 12.3) | 0.34 |
|  | Girl | 1.3  (-0.7, 3.3) |  | 1.8  (-1.6, 5.3) |  | 1.4  (-1.5, 4.5) |  | 0.5  (-1.7, 2.7) |  | -0.03  (-6.5, 6.9) |  |

Abbreviations: TC, total cholesterol; LDL-C, low-density lipoprotein cholesterol; non-HDL-C, non-high-density lipoprotein cholesterol; HDL-C, high-density lipoprotein cholesterol; TG, triglyceride; CI, confidence interval; *p*-int, *p*-value for interaction; PFOA, perfluorooctanoic acid; PFOS, perfluorooctane sulfonic acid; PFHxS, perfluorohexane sulfonic acid; PFNA, perfluorononanoic acid; PFDeA, perfluorodecanoic acid.

The results were estimated from linear regression models with appropriate strata, cluster, and weight variables, adjusted for age, body mass index, paternal educational level, maternal educational level, tobacco smoking, secondhand smoke exposure, alcohol consumption, regular physical exercise, and fish intake. The models that considered urine mercury levels as an exposure were additionally adjusted for urine creatinine levels.

# **Table S8.** Percent changes in blood lipid levels per doubling of concentrations of per- and polyfluoroalkyl substances and heavy metals, estimated from linear regression models not adjusted for body mass index

|  | TC | | LDL-C | | Non-HDL-C | | HDL-C | | TG | |
| --- | --- | --- | --- | --- | --- | --- | --- | --- | --- | --- |
|  | Percent (%) change | 95% CI | Percent (%) change | 95% CI | Percent (%) change | 95% CI | Percent (%) change | 95% CI | Percent (%) change | 95% CI |
| PFOA | 1.8 | -0.2, 3.8 | 3.3 | 0.3, 6.4 | 2.2 | -0.6, 5.1 | 0.8 | -1.5, 3.0 | -1.7 | -6.9, 3.8 |
| PFOS | 1.8 | 0.4, 3.2 | 0.9 | -1.8, 3.7 | 1.4 | -0.5, 3.4 | 2.4 | 0.3, 4.6 | 2.1 | -2.4, 6.8 |
| PFHxS | 0.0 | -1.0, 1.0 | -0.1 | -1.9, 1.8 | -0.1 | -1.7, 1.5 | 0.2 | -1.0, 1.4 | -0.2 | -3.9, 3.6 |
| PFNA | 3.0 | 0.9, 5.2 | 5.4 | 2.0, 8.9 | 3.7 | 0.6, 6.8 | 1.5 | -1.0, 4.1 | -1.8 | -7.6, 4.4 |
| PFDeA | 3.8 | 1.6, 6.0 | 7.5 | 3.7, 11.4 | 4.2 | 0.9, 7.6 | 3.3 | 0.6, 6.0 | -5.9 | -12.7, 1.4 |
| Lead | -0.2 | -1.8, 1.5 | -1.0 | -3.6, 1.6 | -1.4 | -3.8, 1.0 | 2.4 | -0.6, 5.6 | -3.6 | -10.5, 4.0 |
| Mercury | 1.8 | 0.3, 3.3 | 2.6 | 0.0, 5.2 | 3.0 | 0.7, 5.3 | -1.0 | -2.4, 0.5 | 3.5 | -1.0, 8.3 |

Abbreviations: TC, total cholesterol; LDL-C, low-density lipoprotein cholesterol; non-HDL-C, non-high-density lipoprotein cholesterol; HDL-C, high-density lipoprotein cholesterol; TG, triglyceride; CI, confidence interval; PFOA, perfluorooctanoic acid; PFOS, perfluorooctane sulfonic acid; PFHxS, perfluorohexane sulfonic acid; PFNA, perfluorononanoic acid; PFDeA, perfluorodecanoic acid.

The results were estimated from linear regression models with appropriate strata, cluster, and weight variables, adjusted for age, gender, paternal educational level, maternal educational level, tobacco smoking, secondhand smoke exposure, alcohol consumption, regular physical exercise, and fish intake. The models that considered urine mercury levels as an exposure were additionally adjusted for urine creatinine levels.

# **Table S9.** Percent changes in blood lipid levels per doubling of concentrations of per- and polyfluoroalkyl substances and heavy metals, estimated from linear regression models adjusted for the intake of big fish and tuna instead of total fish intake

|  | TC | | LDL-C | | Non-HDL-C | | HDL-C | | TG | |
| --- | --- | --- | --- | --- | --- | --- | --- | --- | --- | --- |
|  | Percent (%) change | 95% CI | Percent (%) change | 95% CI | Percent (%) change | 95% CI | Percent (%) change | 95% CI | Percent (%) change | 95% CI |
| PFOA | 1.6 | -0.5, 3.8 | 3.0 | -0.1, 6.2 | 2.0 | -1.0, 5.1 | 0.8 | -1.3, 3.0 | -1.5 | -7.3, 4.8 |
| PFOS | 1.8 | 0.3, 3.3 | 0.8 | -2.0, 3.6 | 1.6 | -0.4, 3.7 | 1.9 | 0.0, 3.9 | 3.4 | -0.4, 7.4 |
| PFHxS | -0.1 | -1.1, 0.9 | -0.2 | -2.1, 1.7 | -0.2 | -1.9, 1.4 | 0.0 | -1.0, 1.1 | -0.1 | -3.8, 3.7 |
| PFNA | 2.6 | 0.4, 5.0 | 4.6 | 1.2, 8.2 | 3.0 | -0.2, 6.3 | 1.8 | -0.6, 4.2 | -1.8 | -7.8, 4.6 |
| PFDeA | 3.5 | 1.2, 5.9 | 6.6 | 3.0, 10.4 | 4.1 | 0.9, 7.5 | 2.5 | 0.1, 5.0 | -3.2 | -10, 4.2 |
| Lead | -0.2 | -1.8, 1.5 | -1.1 | -3.7, 1.7 | -1.5 | -3.7, 0.8 | 2.5 | -0.3, 5.4 | -3.8 | -10.2, 3.0 |
| Mercury | 1.4 | 0.0, 2.8 | 1.9 | -0.5, 4.3 | 2.2 | 0.1, 4.2 | -0.4 | -1.9, 1.0 | 2.7 | -1.8, 7.3 |

Abbreviations: TC, total cholesterol; LDL-C, low-density lipoprotein cholesterol; non-HDL-C, non-high-density lipoprotein cholesterol; HDL-C, high-density lipoprotein cholesterol; TG, triglyceride; CI, confidence interval; PFOA, perfluorooctanoic acid; PFOS, perfluorooctane sulfonic acid; PFHxS, perfluorohexane sulfonic acid; PFNA, perfluorononanoic acid; PFDeA, perfluorodecanoic acid.

The results were estimated from linear regression models with appropriate strata, cluster, and weight variables, adjusted for age, gender, body mass index, paternal educational level, maternal educational level, tobacco smoking, secondhand smoke exposure, alcohol consumption, regular physical exercise, and intake of big fish and tuna. The models that considered urine mercury levels as an exposure were additionally adjusted for urine creatinine levels.

# **Table S10.** Percent changes in blood lipid levels per doubling of concentrations of per- and polyfluoroalkyl substances and heavy metals, estimated from linear regression models additionally adjusted for frozen meal intake

|  | TC | | LDL-C | | Non-HDL-C | | HDL-C | | TG | |
| --- | --- | --- | --- | --- | --- | --- | --- | --- | --- | --- |
|  | Percent (%) change | 95% CI | Percent (%) change | 95% CI | Percent (%) change | 95% CI | Percent (%) change | 95% CI | Percent (%) change | 95% CI |
| PFOA | 1.8 | -0.3, 3.9 | 3.3 | 0.2, 6.4 | 2.2 | -0.7, 5.2 | 0.8 | -1.4, 3.0 | -1.6 | -7.2, 4.2 |
| PFOS | 1.9 | 0.4, 3.4 | 0.9 | -1.9, 3.8 | 1.8 | -0.2, 3.9 | 1.9 | -0.1, 4.0 | 3.4 | -0.4, 7.4 |
| PFHxS | 0.0 | -1.0, 1.0 | -0.1 | -2.0, 1.8 | 0.0 | -1.6, 1.6 | 0.0 | -1.1, 1.1 | 0.2 | -3.5, 4.0 |
| PFNA | 3.0 | 0.9, 5.2 | 5.3 | 1.8, 8.9 | 3.4 | 0.4, 6.6 | 1.9 | -0.6, 4.5 | -2.6 | -8.2, 3.4 |
| PFDeA | 3.9 | 1.7, 6.3 | 7.5 | 3.6, 11.5 | 4.6 | 1.2, 8.1 | 2.8 | 0.2, 5.5 | -4.5 | -11.1, 2.7 |
| Lead | -0.2 | -1.8, 1.5 | -1.2 | -3.8, 1.5 | -1.5 | -3.6, 0.7 | 2.6 | -0.2, 5.6 | -4.0 | -10.4, 2.9 |
| Mercury | 1.6 | 0.2, 3.0 | 2.4 | -0.1, 4.9 | 2.5 | 0.5, 4.5 | -0.5 | -1.9, 1.0 | 2.1 | -2.1, 6.6 |

Abbreviations: TC, total cholesterol; LDL-C, low-density lipoprotein cholesterol; non-HDL-C, non-high-density lipoprotein cholesterol; HDL-C, high-density lipoprotein cholesterol; TG, triglyceride; CI, confidence interval; PFOA, perfluorooctanoic acid; PFOS, perfluorooctane sulfonic acid; PFHxS, perfluorohexane sulfonic acid; PFNA, perfluorononanoic acid; PFDeA, perfluorodecanoic acid.

The results were estimated from linear regression models with appropriate strata, cluster, and weight variables, adjusted for age, gender, body mass index, paternal educational level, maternal educational level, tobacco smoking, secondhand smoke exposure, alcohol consumption, regular physical exercise, fish intake, and frozen meal intake. The models that considered urine mercury levels as an exposure were additionally adjusted for urine creatinine levels.

# **Table S11.** Associations of urine mercury, blood mercury, and urine cadmium levels with blood lipid levels

|  | TC | | LDL-C | | Non-HDL-C | | HDL-C | | TG | |
| --- | --- | --- | --- | --- | --- | --- | --- | --- | --- | --- |
|  | Percent (%) change | 95% CI | Percent (%) change | 95% CI | Percent (%) change | 95% CI | Percent (%) change | 95% CI | Percent (%) change | 95% CI |
| Urine mercury | 1.8 | 0.3, 3.2 | 2.4 | -0.01, 5.0 | 2.6 | 0.3, 4.9 | -0.2 | -1.7, 1.4 | 1.9 | -2.7, 6.8 |
| Blood mercury | 2.4 | 0.7, 4.1 | 2.3 | -0.6, 5.2 | 4.0 | 1.5, 6.5 | -1.2 | -3.3, 1.1 | 7.0 | 1.6, 12.8 |
| Urine cadmium |  |  |  |  |  |  |  |  |  |  |
| Below the LOD | Ref. |  | Ref. |  | Ref. |  | Ref. |  | Ref. |  |
| LOD–the median | -0.5 | -3.2, 2.3 | 2.7 | -1.9, 7.5 | 0.8 | -2.8, 4.6 | -2.3 | -5.9, 1.5 | -4.0 | -12.7, 5.6 |
| ≥ the median | -0.4 | -3.4, 2.8 | 2.9 | -2.7, 8.9 | 1.1 | -3.5, 5.8 | -3.6 | -6.8, -0.4 | -4.1 | -13.2, 6.1 |

Abbreviations: TC, total cholesterol; LDL-C, low-density lipoprotein cholesterol; non-HDL-C, non-high-density lipoprotein cholesterol; HDL-C, high-density lipoprotein cholesterol; TG, triglyceride; CI, confidence interval; LOD, limit of detection; Ref., reference.

Urine mercury levels were adjusted for urine dilution effects using the conventional standardization method, dividing urine mercury levels by urine creatinine levels. Urine cadmium levels were categorized as below the LOD, greater than or equal to the LOD but below the median, and greater than or equal to the median. The results were estimated from linear regression models with appropriate strata, cluster, and weight variables, adjusted for age, gender, body mass index, paternal educational level, maternal educational level, tobacco smoking, secondhand smoke exposure, alcohol consumption, regular physical exercise, and fish intake. The models that considered urine cadmium levels as an exposure were additionally adjusted for urine creatinine levels.


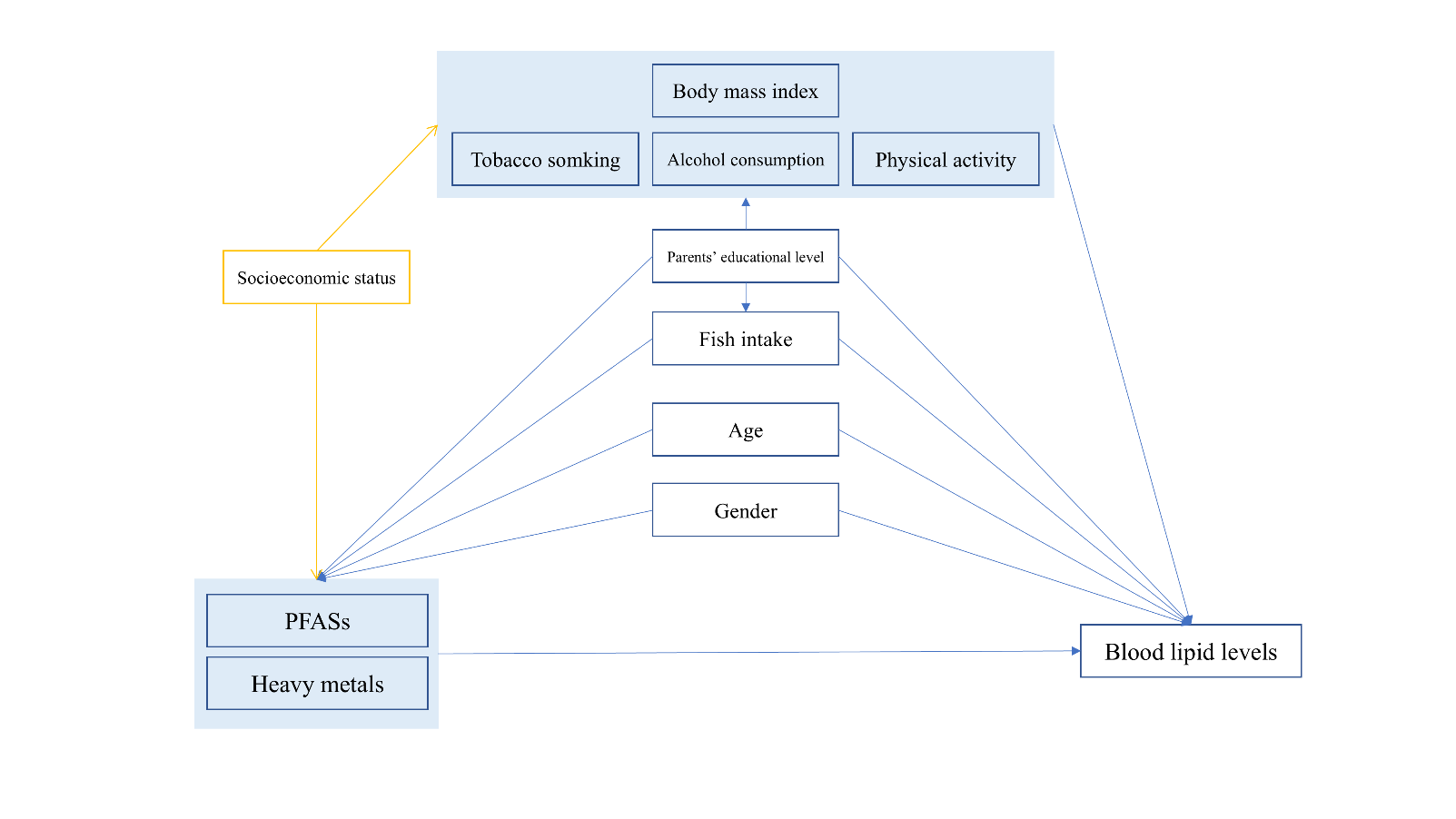


# **Fig. S1.** Assumed causal pathway for the impacts of per- and polyfluoroalkyl substance and heavy metal exposures on blood lipid levels. Abbreviation: PFASs, per- and polyfluoroalkyl substances.


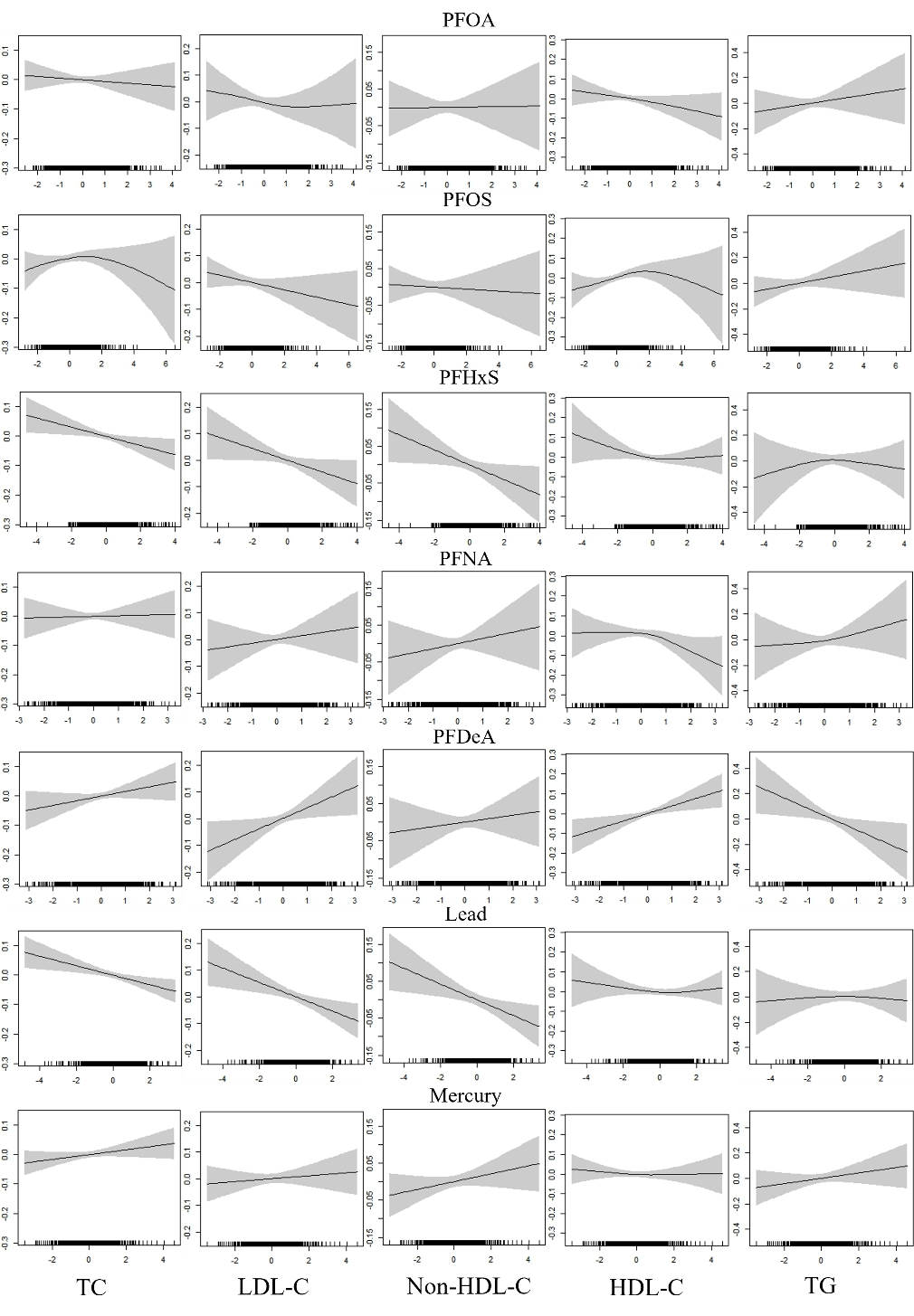


**Fig. S2.** Exposure-outcome relationships between per- and polyfluoroalkyl substances and heavy metals and blood lipid levels, estimated using generalized additive models. Abbreviations: PFOA, perfluorooctanoic acid; PFOS, perfluorooctane sulfonic acid; PFHxS, perfluorohexane sulfonic acid; PFNA, perfluorononanoic acid; PFDeA, perfluorodecanoic acid; TC, total cholesterol; LDL-C, low-density lipoprotein cholesterol; non-HDL-C, non-high-density lipoprotein cholesterol; HDL-C, high-density lipoprotein cholesterol; TG, triglyceride. The models were adjusted for age, gender, body mass index, paternal educational level, maternal educational level, tobacco smoking, secondhand smoke exposure, alcohol consumption, regular physical exercise, fish intake, and urine creatinine levels.


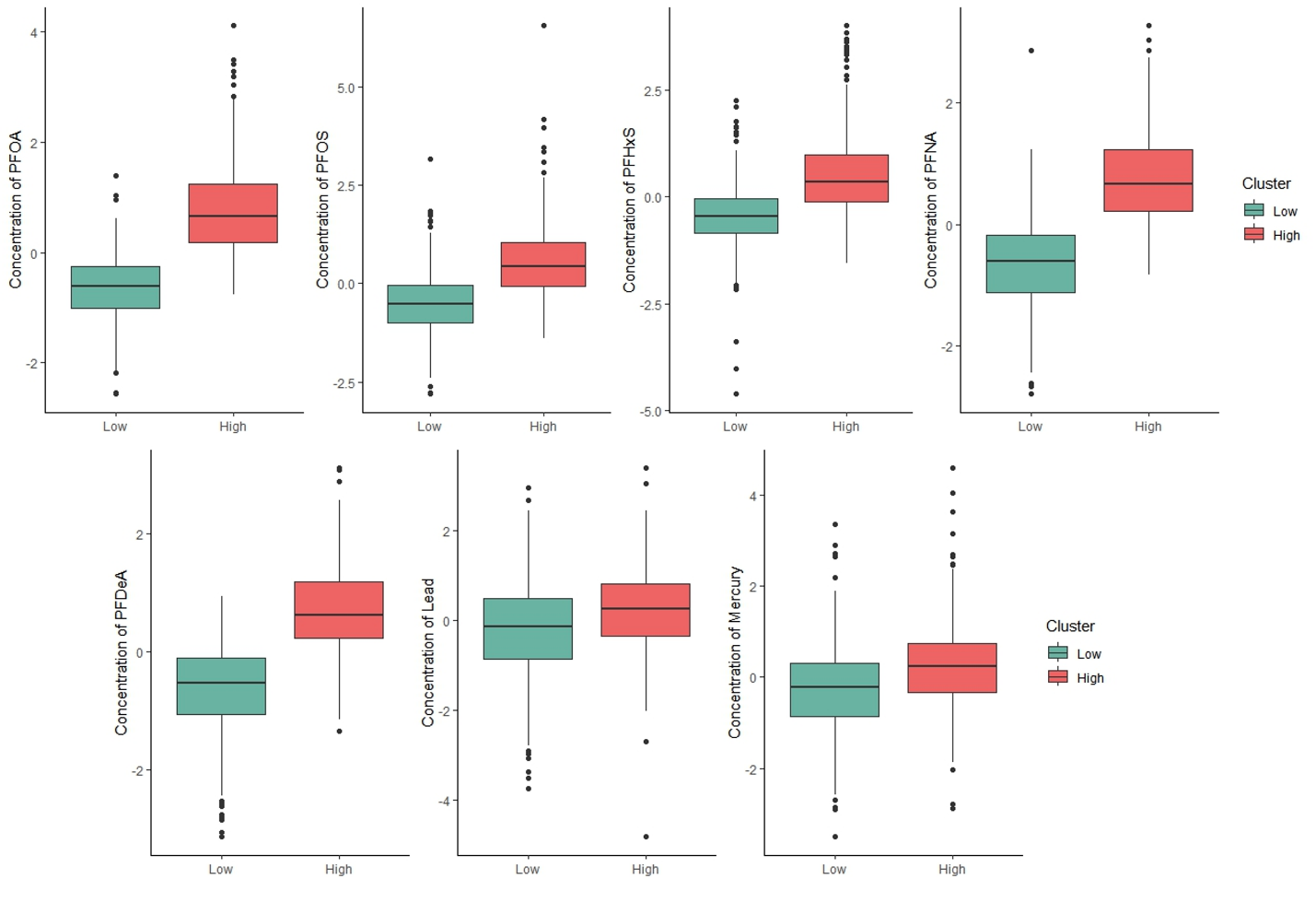


**Fig. S3.** Distributions of concentrations of per- and polyfluoroalkyl substances and heavy metals in high and low pollutant exposure clusters, as identified from the k-means clustering analysis. Abbreviations: PFOA, perfluorooctanoic acid; PFOS, perfluorooctane sulfonic acid; PFHxS, perfluorohexane sulfonic acid; PFNA, perfluorononanoic acid; PFDeA, perfluorodecanoic acid.

# **References**

1. Song X, Ye T, Jing D, Wei K, Ge Y, Bei X, et al. Association between exposure to per- and polyfluoroalkyl substances and levels of lipid profile based on human studies. Rev Environ Health. 2024.

2. Zheng Q, Yan W, Gao S, Li X. The effect of PFAS exposure on glucolipid metabolism in children and adolescents: a meta-analysis. Front Endocrinol (Lausanne). 2024;15:1261008.

3. Kit BK, Kuklina E, Carroll MD, Ostchega Y, Freedman DS, Ogden CL. Prevalence of and trends in dyslipidemia and blood pressure among US children and adolescents, 1999-2012. JAMA Pediatr. 2015;169:272–9.

4. Overview [Internet]. [cited 2024 Jul 26]. Available from: <https://chm.pops.int/Implementation/IndustrialPOPs/PFAS/Overview/tabid/5221/Default.aspx>

5. Zhang Y, Xu C, Fu Z, Shu Y, Zhang J, Lu C, et al. Associations between total mercury and methyl mercury exposure and cardiovascular risk factors in US adolescents. Environ Sci Pollut Res Int. 2018;25:6265–72.

6. Poursafa P, Ataee E, Motlagh ME, Ardalan G, Tajadini MH, Yazdi M, et al. Association of serum lead and mercury level with cardiometabolic risk factors and liver enzymes in a nationally representative sample of adolescents: the CASPIAN-III study. Environ Sci Pollut Res Int. 2014;21:13496–502.

7. Fan Y, Zhang C, Bu J. Relationship between selected serum metallic elements and obesity in children and adolescents in the U.S. Nutrients. 2017;9:104.
